# Circular RNA_LARP4 inhibits cell proliferation and invasion of gastric cancer by sponging miR-424-5p and regulating LATS1 expression

**DOI:** 10.1186/s12943-017-0719-3

**Published:** 2017-09-11

**Authors:** Jing Zhang, Hui Liu, Lidan Hou, Ge Wang, Rui Zhang, Yanxia Huang, Xiaoyu Chen, Jinshui Zhu

**Affiliations:** 1 Department of Gastroenterology, Shanghai Jiao Tong University Affiliated Sixth People’s Hospital, No. 600 Yishan Road, Shanghai, 200233 China; 2 Department of Gastroenterology, Shanghai Ninth People’s Hospital, Shanghai Jiao Tong University School of Medicine, Shanghai, China

**Keywords:** Circular RNA_LARP4, miR-424-5p, LATS1, Invasion, Gastric cancer

## Abstract

**Background:**

Non-coding RNAs (ncRNAs) have been shown to regulate gene expression involved in tumor progression of multiple malignancies. Our previous studies indicated that large tumor suppressor kinase 1 (LATS1), a core part of Hippo signaling pathway, functions as a tumor suppressor in gastric cancer (GC). But, the underlying molecular mechanisms by which ncRNAs modulate LATS1 expression in GC remain undetermined.

**Methods:**

The correlation of LATS1 and has-miR-424-5p (miR-424) expression with clinicopathological characteristics and prognosis of GC patients was analyzed by TCGA RNA-sequencing data. A novel circular RNA_LARP4 (circLARP4) was identified to sponge miR-424 by circRNA expression profile and bioinformatic analysis. The binding site between miR-424 and LATS1 or circLARP4 was verified using dual luciferase assay and RNA immunoprecipitation (RIP) assay. The expression and localization of circLARP4 in GC tissues were investigated by fluorescence in situ hybridization (FISH). MTT, colony formation, Transwell and EdU assays were performed to assess the effects of miR-424 or circLARP4 on cell proliferation and invasion.

**Results:**

Increased miR-424 expression or decreased LATS1 expression was associated with pathological stage and unfavorable prognosis of GC patients. Ectopic expression of miR-424 promoted proliferation and invasion of GC cells by targeting LATS1 gene. Furthermore, circLARP4 was mainly localized in the cytoplasm and inhibited biological behaviors of GC cells by sponging miR-424. The expression of circLARP4 was downregulated in GC tissues and represented an independent prognostic factor for overall survival of GC patients.

**Conclusion:**

circLARP4 may act as a novel tumor suppressive factor and a potential biomarker in GC.

**Electronic supplementary material:**

The online version of this article (10.1186/s12943-017-0719-3) contains supplementary material, which is available to authorized users.

## Background

Gastric cancer (GC) is the fourth common gastrointestinal malignancy and the third leading cause of cancer-related deaths worldwide [1]. Despite a steady decline in GC incidence and mortality rates in recent years due to improved nutritional compositions and *H. pylori* eradication [1], this disease still yields a great threat to human health, leading to a poor prognosis for GC patients, with a 5-year overall survival (OS) rate of less than 30% duo to tumor metastasis and recurrence [2]. Therefore, to discover novel molecular mechanisms and critical signaling pathways, activated or inactivated in GC, is required for developing effective therapeutic strategies for anticancer therapy in GC.

Hippo signaling pathway was previously known to control organ size and growth, and accumulating evidence shows that this pathway acts a pivotal role in the regulation of cell proliferation, metastasis and oncogenesis [3–6]. Large tumor suppressor kinase 1 (LATS1) as a core member of this pathway dominates breast cell fate [7] and modulates liver progenitor cell proliferation and differentiation [8, 9]. Decreased LATS1 expression is associated with unfavorable prognosis and contributes to glioma progression [10]. Our previous study showed that loss of LATS1 is correlated with poor survival and recurrence and promotes growth and metastasis of GC cells [11]. But, LATS1/2 is proved to inhibit tumor immunity and provides a concept for targeting LATS1/2 in cancer immunotherapy [12].

Considerable studies highlight the regulatory mechanisms by which non-coding RNAs (ncRNAs) participate in the development of diseases including cancer [13]. microRNAs (miRNAs), an evolutionarily conserved group of small regulatory ncRNAs, negatively modulate the expression of protein-coding genes [14]. Moreover, some miRNAs are implicated in carcinogenesis by regulating Hippo signaling. For example, miR-130a-YAP positive feedback loop facilitates organ size and tumorigenesis [15], while miR-129 suppresses ovarian cancer survival via repression of Hippo signaling effectors YAP and TAZ [16]. miR-135b, miR-31 and miR-181c function as oncogenes boosting tumor metastasis and chemo-resistance by targeting Hippo signaling members MST1, LATS2, MOB1 and SAV1 [17–19], thereby providing a novel mechanism for Hippo signaling inactivation in cancer.

Circular RNAs (circRNAs) as a novel type of ncRNAs derived from exons, introns or intergenic regions have a covalently closed continuous loop, display cell or tissue-specific expression and are conserved across species due to resistance to RNase R [20, 21], The expression of circRNAs is highly stable in comparison with their linear counterparts, and is predominantly localized in the cytoplasm, indicating important functions for circRNAs in human diseases [22, 23]. Emerging evidence shows that some circRNAs as miRNA sponges modulate gene transcription and interact with RNA binding proteins (RBPs) involved in tumorigenesis [20, 21]. ciRS-7 serves as miR-7 sponge regulating the expression of several oncogenes [24], and circHIPK3 as miR-124 sponge suppresses cell proliferation in multiple caners [25]. circRNA expression profiles reveal a tumor-promoting role of TCF25-miR-103a-3p/miR-107 axis in bladder cancer [26] and circRNA_001569/miR-145 axis in colorectal cancer [27], providing novel promising markers for cancer diagnosis and therapy.

In the present study, we identified an oncogenic miR-424, which was upregulated in GC tissues and was negatively correlated with LATS1 expression. High expression of miR-424 or low expression of LATS1 was closely associated with pathological staging, poor survival and recurrence of GC patients, and miR-424 overexpression promoted cell growth and invasion by targeting LATS1 gene. Furthermore, we characterized a circRNA derived from LARP4 gene locus, termed as circLARP4, which was downregulated in GC tissues, and suppressed cell proliferation and invasion by sponging miR-424 and upregulating LATS1 gene. Therefore, circLARP4 might act as a tumor suppressive factor and an independent prognostic factor for survival of GC patients.

## Methods

### Clinical data

The clinical and pathological data of 387 cases of GC patients and 41 adjacent normal tissues as well as the relative expression levels of LATS1 and miRNAs (has-miR-16-5p, has-miR-15a-5p, has-miR-15b-5p, has-miR-590-3p and has-miR-424-5p) were downloaded from The Cancer Genome Atlas 2015 RNA sequencing database (http://xena.ucsc.edu/getting-started/). The human tissue microarray of 80 paired GC patients (Cat No. STC1602) was purchased from the shanghai Superbiotek Pharmaceutical Technology Co., Ltd. (Shanghai, PR, China). The protocols used in our study were approved by the Ethics Committee of Shanghai Jiao Tong University Affiliated Sixth People’s Hospital. The GC patients’ specimens were classified according to the 2004 WHO criteria and TNM staging system, and clinicopathological characteristics of GC patients from TCGA and tissue microarray were shown in Additional file 1: Table S1–2.

### Identification of miRNAs targeting LATS1 gene in cancer tissues

We identified the miRNAs that target LATS1 gene in cancer by using the StarBase v2.0 (http://starbase.sysu.edu.cn) and the strict screening conditions including two prediction algorithms (Pctar and miRanda), very high stringency (>5) and being expressed in at least three cancer types were limited to predict the miRNAs targeting LATS1 gene.

### Cell culture

Normal human gastric epithelial cell line GES-1 and GC cell lines (SGC-7901, MKN-45, MKN-28, HGC-27, MGC-803, AGS, BGC-823) were from Digestive Disease Laboratory of Shanghai Sixth People’s Hospital. Cells were cultured in Dulbecco’s Modified Eagle medium (DMEM) medium supplemented with 10% heat-inactivated fetal bovine serum (FBS), 100 U/ml of penicillin, and 100 μg/ml of streptomycin (HyClone). Cells in this medium were placed in a humidified atmosphere containing 5% CO_2_ at 37 °C. All cells were used for study within 6 months.

### Quantitative real-time PCR (qRT-PCR)

Total RNA was extracted using TRIzol and reverse transcription was performed using M-MLV and cDNA amplification using the SYBR Green Master Mix kit (Takara, Otsu, Japan). In addition, total RNA was isolated using a High Pure miRNA isolation kit (Roche) and RT-PCR using a TaqMan MicroRNA Reverse Transcription kit (Life Technologies). The nuclear and cytoplasmic fractions were isolated using NE-PER Nuclear and Cytoplasmic Extraction Reagents (Thermo Scientific). The primers were listed in Additional file 1: Table S3.

### Western blotting analysis

HGC-27 and MKN-28 cells were harvested and extracted using lysis buffer (100 mM Tris-HCl, 2% SDS, 1 mM Mercaptoethanol, 25% Glycerol). Cell extracts were boiled in loading buffer and equal amount of cell extracts were separated on 15% SDS-PAGE gels. Separated protein bands were transferred into polyvinylidene fluoride (PVDF) membranes. The primary antibodies-anti-LATS1 (ab70561, Rabbit polyclonal antibody, Abcam, Cambridge, MA, USA), anti-YAP (ab52771, Rabbit monoclonal antibody, Abcam, Cambridge, MA, USA), anti-p-YAP (S127) (ab76252, Rabbit monoclonal antibody, Abcam, Cambridge, MA, USA) and anti-GAPDH (ab153802, Rabbit polyclonal antibody, Abcam, Cambridge, MA, USA) were diluted at a ratio of 1:1000 according to the instructions and incubated overnight at 4 °C. Horseradish peroxidase-linked secondary antibodies were added at a dilution ratio of 1:10,000, and incubated at room temperature for 1 h. The membranes were washed with PBS for three times and the immunoreactive bands were visualized using ECL-PLUS/Kit (GE Healthcare, Piscataway, NJ, USA) according to the kit’s instruction.

### Luciferase reporter assay

HGC-27 and MKN-28 cells were seeded into 96-well plates and were co-transfected with a mixture of 60 ng of firefly luciferase reporter, 6 ng of pRL-CMV Renilla luciferase reporter, and miR-424 mimic or inhibitor. After 48 h of incubation, the firefly and Renilla luciferase activities were measured with a dual-luciferase reporter assay (Promega, Madison, WI, USA).

### Plasmid, siRNAs and miRNA mimic and inhibitor

Plasmid mediated LATS1 or circLARP4 overexpression vector, siRNA targeting LATS1 or circLARP4 vector, miR-424 mimic and inhibitor were purchased from Genechem (Shanghai, PR, China) and an empty vector used as a control. The siRNA sequences were shown as below, si-LATS1: ATCCTCGACGAGAGCAGA and si-circLARP4: GGGCAGGCTCCCTTTCCCAAT. HGC-27 and MKN-28 cells were planted in 6-well plates 24 h prior to si-LATS1, si-circLARP4, miR-424 mimic or inhibitor transfection with 50–60% confluence, and then were transfected with Lipofectamine 2000 (Invitrogen, Carlsbad, CA, USA) according to the manufacture instructions.

### Cell viability and transwell invasion assays

Cell viability and Transwell assays were performed as previously described [11].

### Colony formation assay

HGC-27 and MKN-28 cells were trypsinized, and 1 × 10^3^ cells were plated in 6-well plates and incubated at 37 °C for 7 days. Colonies were dyed with dyeing solution containing 0.1% crystal violet and 20% methanol. Cell colonies were then counted and analyzed.

### circRNA microarray analysis

Total RNA from three GC and adjacent normal tissues was quantified using the NanoDrop ND-1000. The sample preparation and microarray hybridization were performed based on the Arraystar’s standard protocols. Briefly, total RNAs were digested with RNase R to eliminate linear RNAs and enrich circular RNAs. Then, the enriched circular RNAs were amplified and transcribed into fluorescent cRNA utilizing a random priming method (Arraystar Super RNA Labeling Kit; Arraystar). The labeled cRNAs were hybridized onto the Arraystar Human circRNA Array (8x15K, Arraystar). After having washed the slides, the arrays were scanned by the Agilent Scanner G2505C.

### Actinomycin D and RNase R treatment

Transcription was prevented by the addition of 2 mg/ml Actinomycin D or DMSO (Sigma-Aldrich, St. Louis, MO, USA) as the negative control. Total RNA (2 μg) was incubated for 30 min at 37 °Cwith 3 U/μg of RNase R (Epicentre Technologies, Madison, WI, USA). After treatment with Actinomycin D and RNase R, the RNA expression levels of LARP4 and circLARP4 were detected by qRT-PCR.

### 5-Ethynyl-20-deoxyuridine (EdU) incorporation assay

The EdU assay was carried out with a Cell-Light EdU DNA Cell Proliferation Kit (RiboBio, Shanghai, PR, China). 1 × 10^4^ cells were seeded in 96-well plate. After incubation with 50 mM EdU for 2 h, the cells were fixed in 4% paraformaldehyde and stained with Apollo Dye Solution. Hoechst-33,342 was used to stain the nucleic acid within the cells. Images were acquired with an Olympus FSX100 microscope (Olympus, Tokyo, Japan), and the percentage of EdU-positive cells was calculated.

### RNA immunoprecipitation (RIP)

RIP assay was carried out by using a Magna RIP RNA-Binding Protein Immunoprecipitation Kit (Millipore) according to the manufacturer’s instructions. Antibodies for RIP assays against AGO2 and IgG were purchased from Abcam (ab5072, Rabbit polyclonal antibody, Cambridge, MA, USA).

### RNA fluorescence in situ hybridization (FISH)

Oligonucleotide modified probe sequence for human circLARP4 (CCATTGGGAAAGGGAGCCTGCCCTACCATAGTCC) was applied for FISH. First, the probe of circLARP4 was marked with DIG-UTP (Roche, 11,209,256,910) for RNA labeling. The cell suspension was pipetted onto autoclaved glass slides, which were washed with PBS and fixed in 4% paraformaldehyde. After dehydration with 70, 95 and 100% ethanol, hybridization was carried out at 37 °C overnight in a dark moist chamber. After hybridization, slides were washed three times in 50% 60 ml formamide/2X SSC for 5 min, and was incubated with anti-DIG-HRP(PerkinElmer, NEF832001EA)at 4 °C overnight, After being washed for 3 times for 10 min at room temperature, the slides were incubated with TSA fluorescent signal reaction solution(PerkinElmer, NEL701001KT, TSA Fluorescein system)for 30 min and was sealed with tablets containing DAPI. The images were acquired using a fluorescence microscopy (Leica, SP8 laser confocal microscopy). The analysis software Image-pro plus 6.0 (Media Cybernetics, Inc., Rockville, MD, USA) was applied to acquire the Immunofluorescence Accumulation Optical Density (IOD) for evaluating the expression level of circLARP4 in GC tissues.

### Statistical analysis

Statistical analyses were carried out by using SPSS 20.0 (IBM, SPSS, Chicago, IL, USA) and GraphPad Prism. Student’s t-test or Chi-square test was used to assess the statistical significance for comparisons of two groups. The Pearson’s correlation coefficient analysis was used to analyze the correlations. Overall survival (OS) was defined as the interval between the dates of surgery and death and OS and disease-free survival (DFS or recurrence) curves were analyzed with the Kaplane-Meier method and log-rank test. Univariate analysis and multivariate models were performed by using a Cox proportional hazards regression model. Receiver operating characteristic (ROC) curves were obtained using cutoff finder online software (http://molpath.charite. de/cutoff/load.jsp). *P* < 0.05 was considered statistically significant.

## Results

### MiR-424-5p was negatively correlated with LATS1 expression in GC

**Fig. 1:**
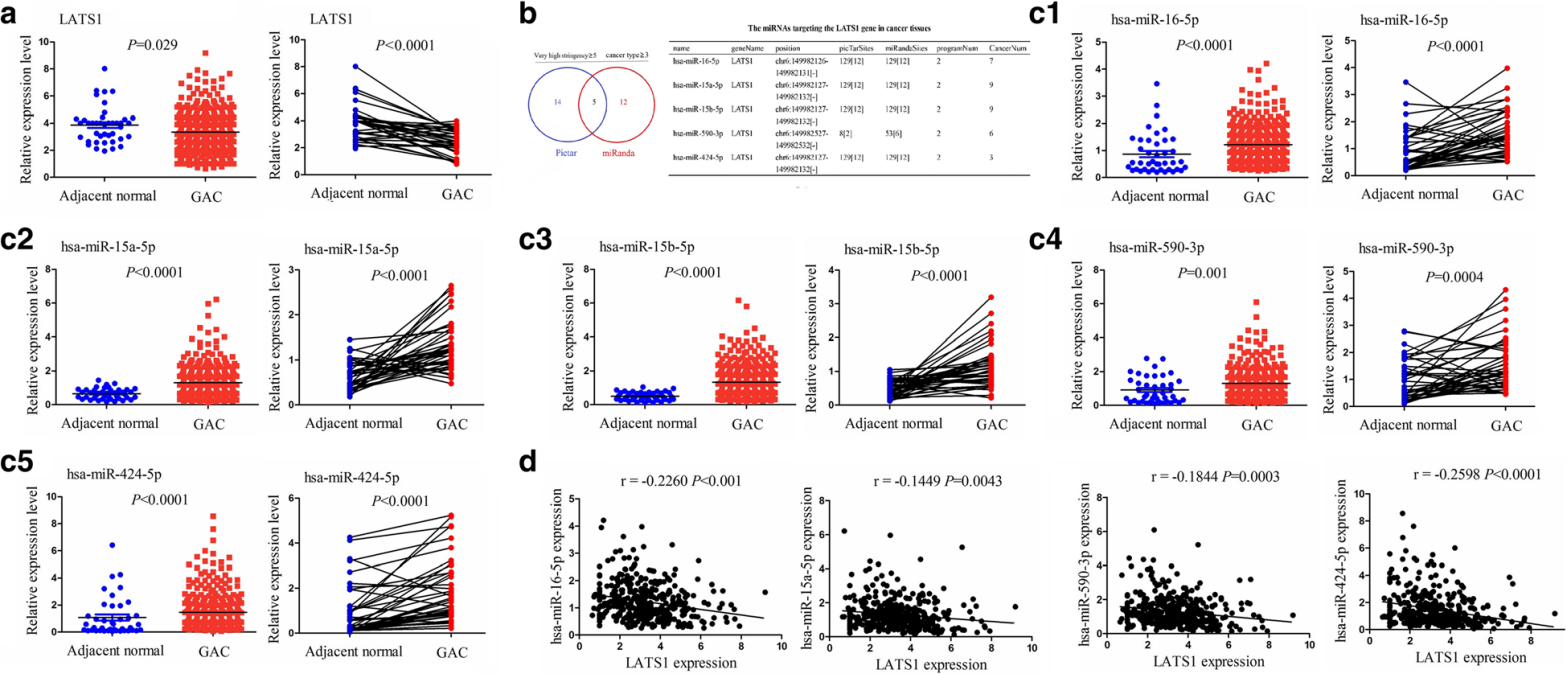
MiR-424-5p was negatively correlated with LATS1 expression in GC. **a** TCGA RNA sequencing database analysis of the relatively differential expression level of LATS1 in human GC and pair-matched normal tissues. **b** Bioinformatic software identification of the miRNAs which target LATS1 gene in human cancer tissues. **c1–5** TCGA analysis of the relatively differential expression levels of miR-16-5p, miR-15a-5p, miR-15b-5p, miR-590-3p and miR-424-5p between human GC and pair-matched normal tissues. **d** Pearson correlation analysis of the correlation of miR-16-5p, miR-15a-5p, miR-590-3p or miR-424-5p with LATS1 expression in human GC tissues

Our previous studies showed that LATS1 is downregulated in GC compared with the pair-matched normal tissues [11]. We further validated a decreased expression of LAST1 in human gastric adenocarcinoma (GAC) tissues (*n* = 387) and adjacent normal tissues (*n* = 41) as well as in paired GAC tissues (n = 41) by using The Cancer Genome Atlas (TCGA) sequencing data (Fig. 1a). To dissect the molecular mechanism of LATS1 downregulation in GC, we first assessed the genetic or epigenetic dysregulation of LATS1 in GC. But, we discovered little evidence regarding the dysregulation of LATS1 at the genetic (Additional file 2: Figure S1a and b) and methylation levels (Additional file 2: Figure S1c), suggesting that genetic alterations (amplification, deletion, mutation and copy number deletion) and methylation modification were not the main cause of the downregulation of LATS1 in GC. Numerous studies show that miRNAs exhibit a pivotal role in GC development by repressing the expression of their target genes [14], indicating that LATS1 might be regulated by miRNAs in GC. Then, we utilized the StarBase v2.0, Pictar and miRanda software to forecast the potential miRNAs that target LATS1 gene 3′ UTR, and identified 5 miRNAs that could bind to LATS1 gene 3′ UTR with very high stringency (Fig. 1b). Furthermore, we investigated the expression levels of these miRNAs in GAC, which were highly expressed in GAC (*n* = 387) compared with adjacent normal tissues (*n* = 41) as well as in paired GAC (n = 41) (Fig. 1c1–5). The correlation analysis showed that, except for the has-miR-15b-5p (Additional file 2: Figure S1d), the other miRNAs displayed the negative correlation with LATS1 expression, of which miR-424-5p (miR-424) had the strongest correlation with LATS1 expression (*r* = −0.2598, *P* < 0.0001) and was selected for further observation.

### MiR-424 and LATS1 expression were associated with clinicopathological characteristics and prognosis of GC patients

**Fig. 2:**
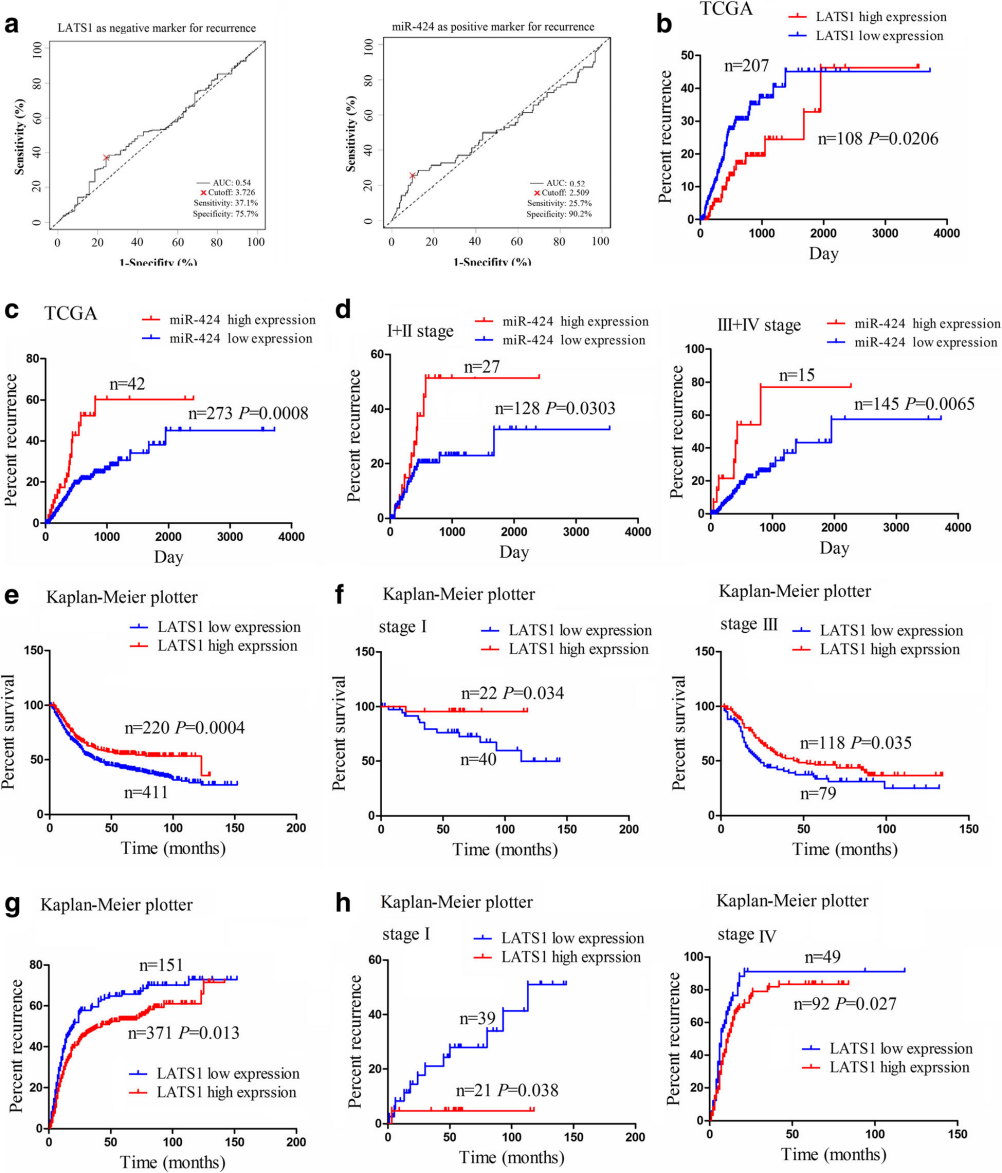
Correlation of LATS1 and miR-424 expression with clinicopathological characteristics and prognosis of GC patients. **a** Receiver operating characteristic (ROC) curve analysis of the cutoff value, sensitivity, specificity and AUC of LATS1 and miR-424 in GC patients. **b** and **c** Kaplan Meier analysis of the correlation of LATS1 and miR-424 expression levels with the recurrence of GC patients. **d** Kaplan Meier analysis of the correlation of miR-424 expression level with the recurrence of GC patients with early stage (stage I + II) and late stage (stage III + IV). Online bioinformatics tool Kaplan-Meier plotter analysis of the correlation of LATS1 expression level with **e** overall survival (OS) and **g** recurrence of GC patients. **f** Kaplan-Meier plotter analysis of the correlation of LATS1 expression level with OS of GC patients with stage I and stage III. **h** Kaplan-Meier plotter analysis of the correlation of LATS1 expression level with recurrence of GC patients with stage I and stage IV

To evaluate whether the expression levels of LATS1 and miR-424 were associated with clinical and pathological characteristics and prognosis of GC patients, as shown in Fig. 2a, based on the cutoff values of LATS1 and miR-424, which were obtained according to their expression levels, OS time and OS status in GAC tissues, the 315 GAC patients were divided into two groups: LATS1 high expression and LATS1 low expression or miR-424 high expression and miR-424 low expression. Receiver operating characteristic (ROC) curve and area under curve (AUC) were determined to assess the sensitivity and specificity of the survival prediction. The sensitivity, specificity and AUC of LATS1 were 37.1%, 75.7% and 0.54 and those of miR-424 were 25.7%, 90.2% and 0.52, indicating that LATS1 and miR-424 might be the potential biomarkers for OS of GAC patients.

As indicated in Additional file 1: Table S4, LATS1 low expression or miR-424 high expression was positively correlated with pathological stage (*P* = 0.004*; P* = 0.036), but harbored no association with other clinical parameters (*P* > 0.05). Kaplan Meier analysis illustrated that GAC patients with LATS1 low expression or miR-424 high expression developed more frequent recurrence (Fig. 2b and c), but had no significant difference in OS compared with the patients with LATS1 high expression or miR-424 low expression (Additional file 3: Figure S2a and b). According to the pathological stage, the patients of early stage (stage I + II) or late stage (stage III + IV) with miR-424 high expression possessed the higher recurrence rate compared with those with miR-424 low expression (Fig. 2d), but the patients of early stage (stage I + II) or late stage (stage III + IV) with LATS1 low expression had no obvious difference in tumor recurrence compared with those with LATS1 high expression (Additional file 3: Figure S2c). To define whether LATS1 or miR-424 expression was independent of other risk factors related to the recurrence of GAC, multivariate analyses were performed by using a Cox proportional hazard model. As shown in Additional file 1: Table S5, 6, in the univariate analysis, gender, LATS1 low expression and miR-424 expression were associated with recurrence of GC patients, but in the final multivariate Cox regression model, it was miR-424 not LATS1 expression that represented an independent prognostic factor for recurrence of GAC.

Furthermore, by using the online bioinformatics tool Kaplan-Meier plotter [28], we found that GC patients with LATS1 low expression developed poorer survival (Fig. 2e) and more frequent recurrence (Fig. 2g). In addition, the patients of stage I or stage III with LATS1 low expression had poorer survival (Fig. 2f) and those of stage I or stage IV harbored higher recurrence (Fig. 2h) compared with those with LATS1 high expression, but the patients with LATS1 low expression had no significant difference in OS for stage II or stage IV (Additional file 3: Figure S2d) and in recurrence for stage II or stage III (Additional file 3: Figure S2e) compared with those with LATS1 high expression.

### LATS1 Was validated as a target gene of miR-424

**Fig. 3:**
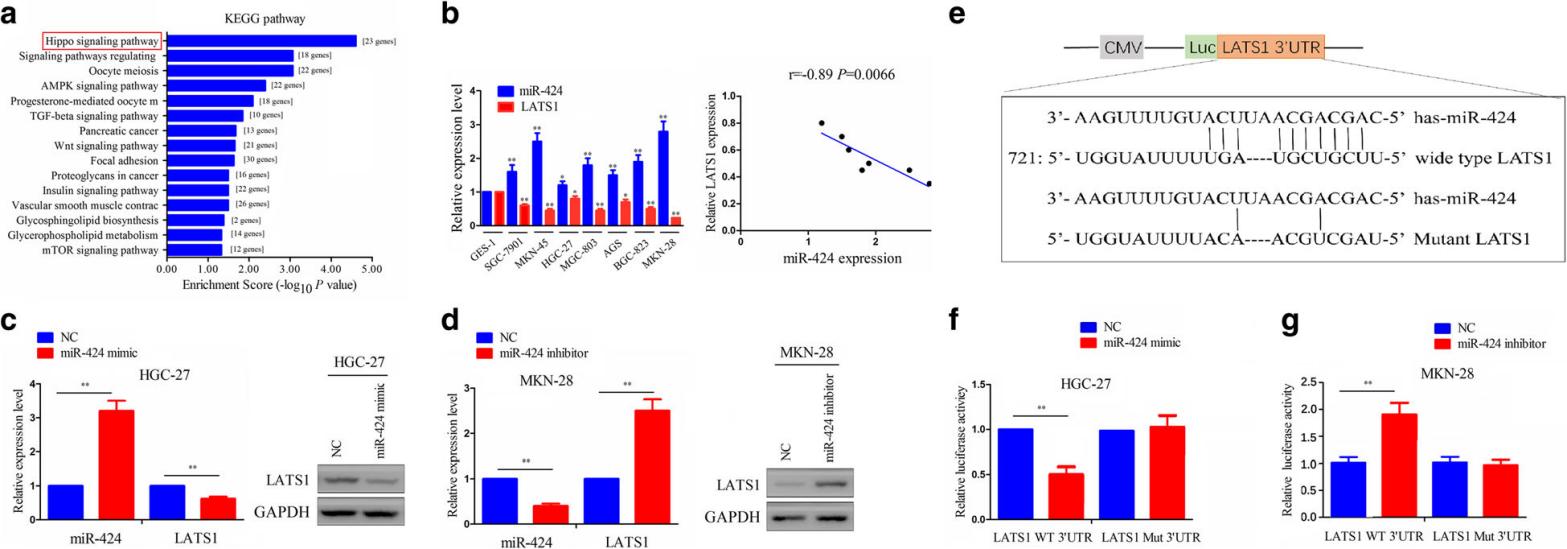
Validation of LATS1 as a target gene of miR-424 in GC cells. **a** KEGG enrichment analysis of the association of miR-424 expression with enriched signaling pathways by using mirPath v.3 software. **b** Relative expression levels of miR-424 and LATS1 in different GC cell lines and GES-1 cells and spearman correlation analysis of their correlation in GC cells. **c** and **d** qRT-PCR and western blot analysis of the expression levels of miR-424 and LATS1 after transfection with miR-424 mimic or inhibitor in HGC-27 or MKN28 cell lines. **e** The binding sites of wild type or mutant LATS1 3’UTR with miR-424. **f** and **g** The luciferase activity of wild type LATS1 3’UTR or mutant LATS1 3’UTR after transfection with miR-424 mimic or inhibitor in HGC-27 or MKN28 cell lines. Data are the means ± SEM of three experiments. **P < 0.01

Having confirmed the negative correlation of miR-424 with LATS1 expression in GC tissues, we were curious about whether LATS1 was a target gene of miR-424 in GC cells. We first utilized the mirPath v.3 software to verify whether miR-424 was related with LATS1 signaling pathway. Consequently, KEGG enrichment analysis demonstrated that miR-424 was principally enriched by Hippo signaling pathway in which LATS1 was a nuclear member (Fig. 3a). Then, we examined the expression levels of miR-424 and LATS1 in GC cell lines by qRT-PCR, which demonstrated that LATS1 had lower expression, while miR-424 exhibited higher expression and negative correlation with LATS1 expression in all GC cell lines compared with the GES-1, of which MKN-28 cell line had the relatively higher miR-424 expression level but HGC-27 cell line had the relatively lower miR-424 expression level (Fig. 3b). Thus, miR-424 mimic (60 μM) and miR-424 inhibitor (80 μM) were respectively transfected into HGC-27 cells with miR-424 low expression and MKN-28 cells with miR-424 high expression. After transfection for 48 h, qRT-PCR and western blot analysis displayed substantially increased expression of miR-424 and decreased expression of LATS1 in miR-424 mimic group in HGC-27 cells (Fig. 3c), and indicated noticeably decreased expression of miR-424 and increased expression of LATS1 in miR-424 inhibitor group in MKN-28 cells (Fig. 3d). Luciferase reporter vectors containing the wild type or mutant LATS1 3’UTR (Fig. 3e) and miR-424 mimic or inhibitor were co-transfected into HGC-27 or MKN-28 cells. Intriguingly, the luciferase activity of wild type LATS1 3’UTR evidently decreased in miR-424 mimic group in HGC-27 cells (Fig. 3f), but increased in miR-424 inhibitor group in MKN-28 cells compared with the NC group (Fig. 3g). However, the luciferase activity of mutant LATS1 3’UTR had no difference between miR-424 mimic or inhibitor group and NC group.

### Ectopic expression of miR-424 promotes GC cell growth and invasion via targeting LATS1 gene

**Fig. 4:**
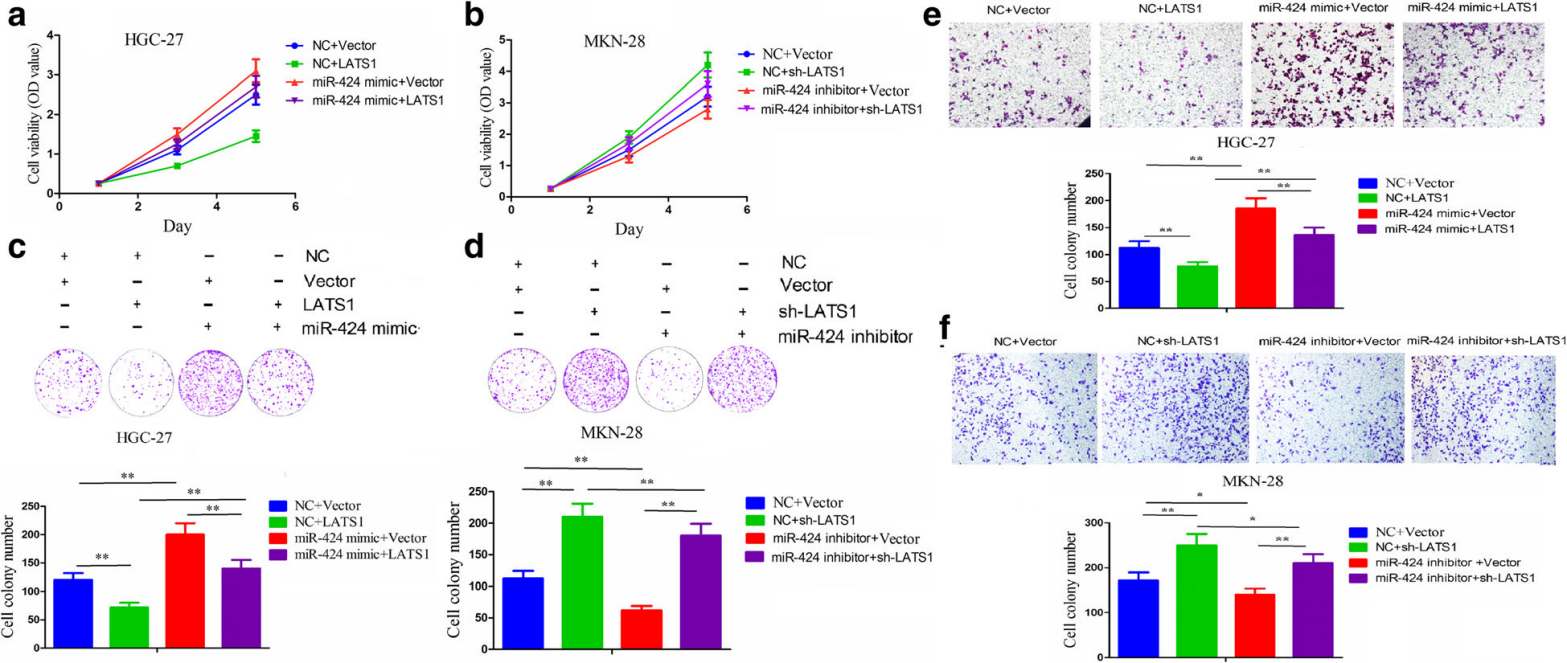
Ectopic expression of miR-424 promotes GC cell growth and invasion by targeting LATS1 gene. **a**, **b** Cell proliferation activity, **c**, **d** colony formation capacity and **e**, **f** invasive potential were respectively measured by MTT, cell colony formation and Transwell assays after transfection with miR-424 mimic+LATS1 overexpression vector or miR-424 inhibitor+sh-LATS1 vector in HGC-27 or MKN-28 cell lines. Data are the means ± SEM of three experiments. *P < 0.05; **P < 0.01

To further observe the effects of miR-424 on LATS1 expression in GC cells, we performed the functional experiments such as MTT, cell colony formation and Transwell assays. First, the expression level of LATS1 was examined after transfection with miR-424 mimic and (or) LATS1 in HGC-27 cells, and miR-424 inhibitor and (or) sh-LATS1 in MKN-28 cells indicated by qRT-PCR (Additional file 4: Figure S3a). Then, the promoting effects of miR-424 mimic on cell proliferation (Fig. 4a), colony formation (Fig. 4c) and invasive potential (Fig. 4e) were reversed by co-transfection of LATS1 overexpression vector in HGC-27 cells, but the inhibitory effects of miR-424 inhibitor on cell proliferation (Fig. 4b), colony formation (Fig. 4d) and invasive potential (Fig. 4f) were rescued by co-transfection of LATS1 shRNA vector in MKN-28 cells. These results suggested that miR-424 might promote GC growth and invasion by targeting LATS1 gene.

### Identification and characteristics of circLARP4 in GC cells

**Fig. 5:**
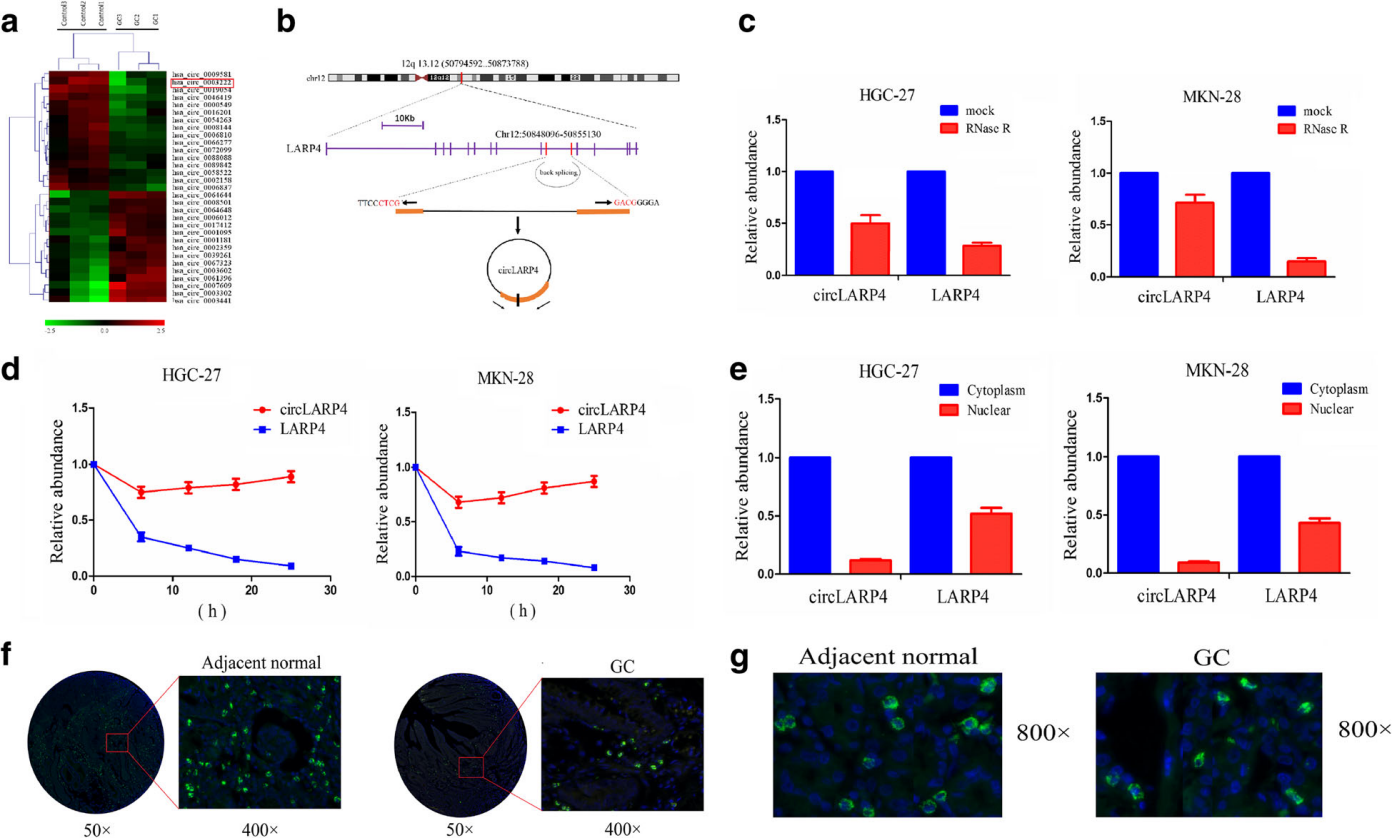
Identification and characteristics of circLARP4 in GC cells. **a** CircRNA microarray chip was used to identify the differentially expressed circRNAs between GC and adjacent normal tissues. **b** The genomic loci of the LATP4 gene and circLARP4. Arrows represent divergent primers that bind to the genomic region of circLARP4. **c** qRT-PCR analysis of circLARP4 and LARP4 RNA after treatment with RNase R in HGC-27 and MKN-28 cells. **d** qRT-PCR analysis of circLARP4 and LARP4 RNA after treatment with Actinomycin D at the indicated time points in HGC-27 and MKN-28 cells. **e** qRT-PCR analysis of circLARP4 and LARP4 RNA in the cytoplasm or the nucleus in HGC-27 and MKN-28 cells. **e** The expression level of circLARP4 was decreased in GC tissues compared the normal tissues. **f** The cellular localization of circLARP4 in GC tissue cells and adjacent normal tissue cells. The nuclei were stained with DAPI for blue color, and the cytoplasmic circLARP4 was stained for green color

Mounting evidence shows that circRNAs as a novel type of ncRNAs sponge miRNAs, regulate gene transcription and interact with RBPs involved in tumorigenesis [20, 21]. To identify the circRNAs that sponge miR-424 in GC, we applied the circRNA expression profile and circBase and microRNA.org software to screen out 30 circRNAs that could sponge and bind to the miR-424 (Fig. 5a, Additional file 1: Table S7). Given the upregulation of miR-424 in GC, 15 circRNAs that were downregulated in GC were selected for further analysis (Fig. 5a, Additional file 1: Table S7). According to the restrictive conditions (*P* < 0.001, Fold change > 2), among 15 circRNAs, only has_circ_101057 conformed to this requirement (Additional file 1: Table S7). We noted that circ_101057 (chr12:50,848,096–50,855,130, Fig. 5b) is derived from exon 9, 10 regions within La ribonucleoprotein domain family member 4 (LARP4) locus, which is located on chromosome 12q13.12. We termed circ_101057 as circLARP4, whose genomic sequence is 7034 nt and spliced mature sequence length is 317 nt. The genomic position reveals that the ninth and tenth exons from the LARP4 gene are intermediated by long introns (Fig. 5b). According to the qRT-PCR analysis, compared with the linear LARP4, circLARP4 gave rise to resistance to digestion induced by RNase R exonuclease, indicating that circLARP4 harbors a loop structure (Fig. 5c). We next observed the stability and localization of circLARP4. After treatment with Actinomycin D, an inhibitor of transcription at the indicated time points, total RNA was separated from HGC-27 and MKN-28 cells. As a result, qRT-PCR analysis showed that the transcript half-life of circLRP4 exceeded 24 h, while that of linear LARP4 displayed about 6 h in HGC-27 and MKN-28 cells (Fig. 5d), indicating that circLARP4 is highly stable in GC cells.

Cytoplasmic and nuclear RNA analysis by qRT-PCR showed that circLARP4 was preferentially localized in the cytoplasm in HGC-27 and MKN-28 cells (Fig. 5e). Furthermore, we utilized FISH to assess circLARP4 expression level and localization in GC tissue cells, and found that circLARP4 had low expression in GC tissues compared with the adjacent normal (Fig. 5f) and the green fluorescent distribution position indicated that circLARP4 was mainly localized in the cytoplasm of both GC and normal tissue cells (Fig. 5g). Collectively, these results suggest that circLARP4 is a highly stable and cytoplasmic circRNA derived from the LARP4 gene locus.

### The effects of circLARP4 on GC cell proliferation and invasion in vitro

**Fig. 6:**
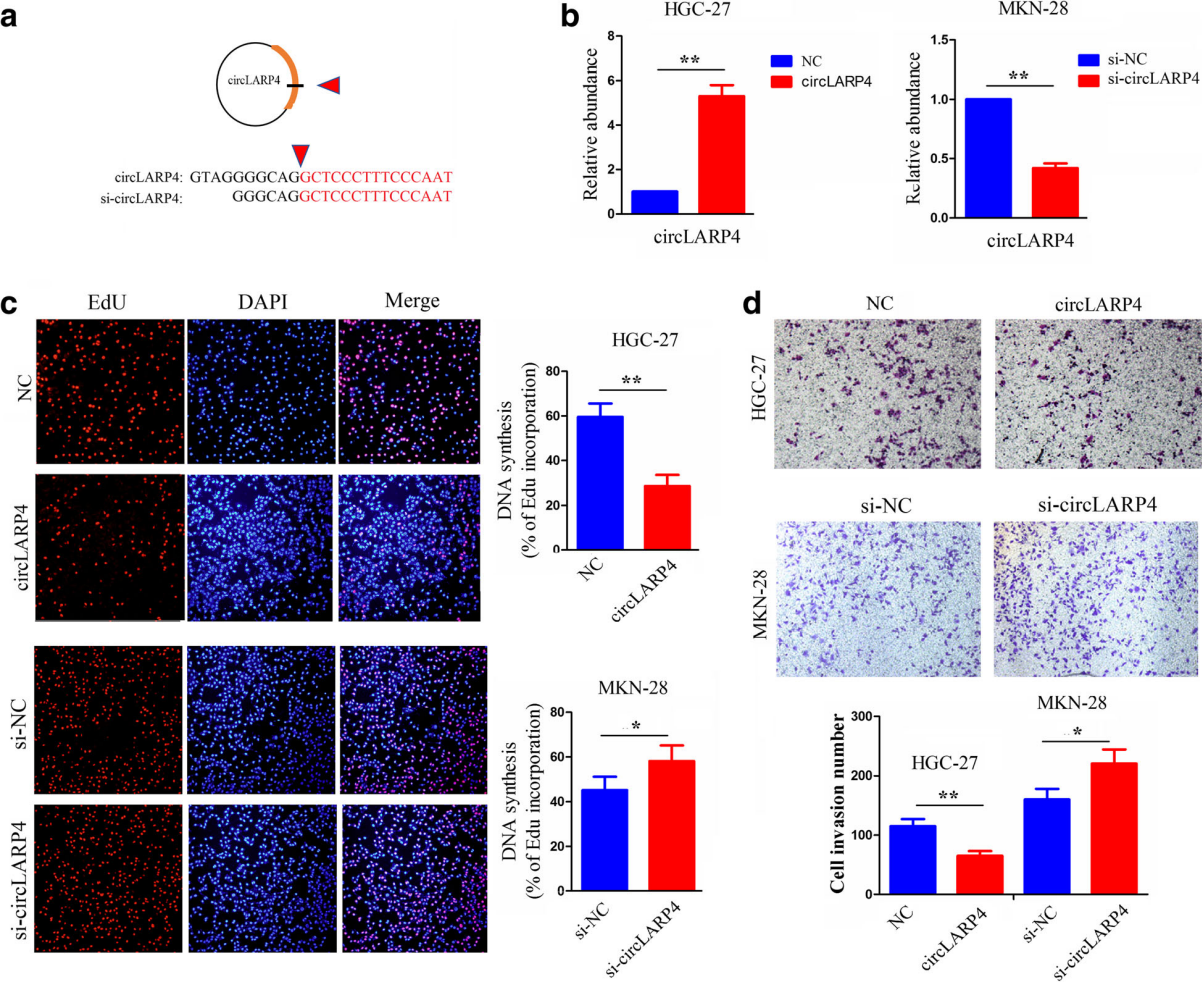
The effects of circLARP4 on GC cell proliferation and invasion. **a** Schematic representation of target sequences of the siRNAs specific to the back-splicing junction of circLARP4. **b** qRT-PCR analysis of the transfection efficiency of circLARP4 overexpression or si-circLARP4 vectors after transfection for 48 h in HGC-27 or MKN-28 cells. **c** Observation of DNA synthesis of HGC-27 or MKN-28 cells transfected with circLARP4 overexpression or si-circLARP4 vectors by EdU assay. **d** Determination of cell invasive potential of HGC-27 or MKN-28 cells transfected with circLARP4 overexpression or si-circLARP4 vectors by Transwell assay. Data are the means ± SEM of three experiments. *P < 0.05; **P < 0.01

The special characteristics of circLARP4 spurred our interest in assessing the biological functions of circLARP4 in GC cells. We first detected the expression level of circLARP4 in GC cell lines by qRT-PCR and found circLARP4 was downregulated in GC cell lines compared with GES-1 cells but had no correlation with the expression of miR-424 and LATS1 in GC cells (Additional file 4: Figure S3b). Then, we designed the circLARP4 overexpression and interference sequences against the back-splicing sequence of circLARP4 (Fig. 6a). After circLARP4 overexpression or siRNA vector was respectively transfected into HGC-27 and MKN-28 cells for 48 h, their transfection efficiency was determined as shown in Fig. 6b. Subsequent cell proliferation and colony formation assays revealed that ectopic expression of circLARP4 inhibited cell growth and colony-forming capacity of GC cells, while knockdown of circLARP4 reversed these effects (Additional file 4: Figure S3c, d). EdU incorporation assay showed that the proliferation of HGC-27 cells was impaired by transfection with circLARP4 overexpression vector, but was strengthened by knockdown of circLARP4 (Fig. 6c). Transwell invasion assay demonstrated that cell invasive potential was weakened by circLARP4 overexpression, but was enhanced by knockdown of circLARP4 (Fig. 6d). Taken together, these results imply that that circLARP4 may be a tumor suppressive factor in GC cells.

### circLARP4 Acts as a miRNA sponge for miR-424 in GC cells

**Fig. 7:**
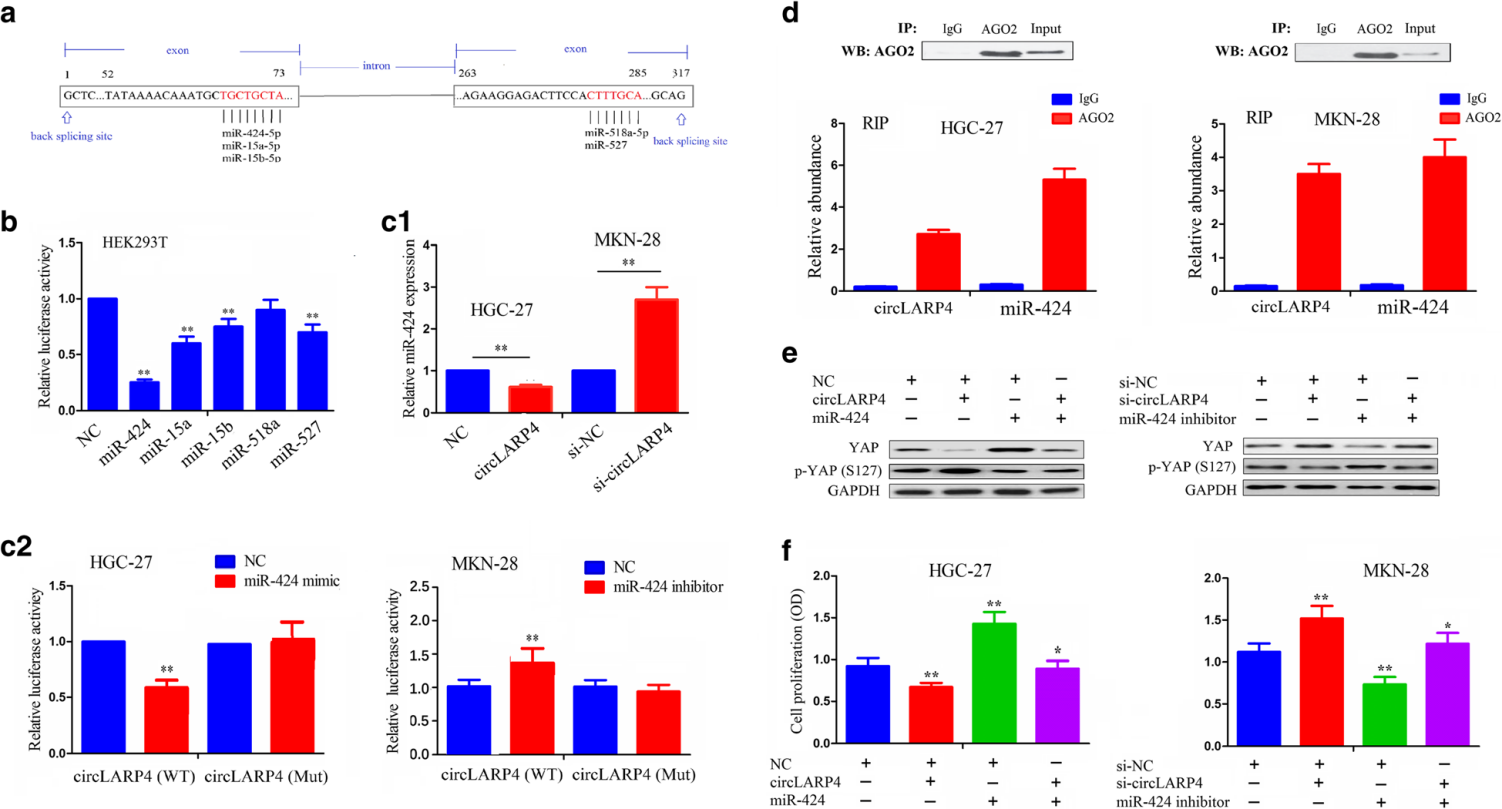
circLARP4 acts as a miRNA sponge for miR-424 in GC cells. **a** Schematic representation of potential binding sites of miRNAs with circLARP4. **b** The luciferase activity of circLARP4 after transfection with pMIR-Luc-circLARP4 combined with each miRNA mimic in HEK-293 T cells. **c1** The effects of circLARP4 overexpression of knockdown on the expression level of miR-424 in HCG-27 or MKN-28 cell line indicated by qRT-PCR. **c2** The luciferase activity of wild type circLARP4 3’UTR or mutant circLARP4 3’UTR after transfection with miR-424 mimic or inhibitor in HGC-27 or MKN-28 cell lines. **d** AGO2 RNA immunoprecipitation (RIP) assay for the amount of circLARP4 and miR-424 in HGC-27 or MKN-28 cells. Ago2 was detected using IP-western blot (up panel), and circLARP4 and miR-424 expression levels were detected using qRT-PCR (down panel). **e** Western blot analysis of the expression levels of YAP and p-YAP (S127) after transfection with circLARP4 + miR-424 in HGC-27 cells or si-circLARP4 + miR-424 inhibitor in MKN-28 cells. **f** MTT analysis of cell proliferation of HGC-27 or MKN-28 cells after transfection with circLARP4 + miR-424 or si-circLARP4 + miR-424 inhibitor. Data are the means ± SEM of three experiments. *P < 0.05; **P < 0.01

Given that circRNAs can act as miRNAs sponge and circLARP4 is stable in the cytoplasm, we first applied the ComiR prediction tool as well as the circLARP4 genomic sequence to predict 5 miRNAs that have the potential to bind to circLARP4 (Fig. 7a and Additional file 5: Figure S4a). We constructed a circLARP4 fragment and incorporated it into downstream of the luciferase reporter gene, and hypothesized that circLARP4 related 5 miRNAs could reduce the luciferase activity of circLARP4. We then carried out a luciferase assay using these 5 miRNAs. The luciferase reporter vector for pMIR-Luc-circLARP4 was co-transfected with each miRNA mimic into HEK-293 T cells. Compared with the negative control (NC), miR-424 lowered the luciferase reporter activity by approximately 75% (Fig. 7b), indicating that miR-424 might have the greater potential to bind to circLARP4 compared with other 4 miRNAs. Afterwards, we examined the mutual regulation between circLARP4 and miR-424 by qRT-PCR analysis, which showed that circLARP4 overexpression reduced the expression level of miR-424 in HCG-27 cells, while circLARP4 knockdown increased the expression level of miR-424 in MKN-28 cells (Fig. 7c1), but miR-424 mimic or inhibitor had no influence on the expression of circLARP4 (Additional file 5: Figure S4b). Luciferase reporter vectors containing the wild type or mutant circLARP4 (Additional file 5: Figure S4c) and miR-424 mimic were co-transfected into HGC-27 and MKN-28 cells. Interestingly, the luciferase activity of wild type circLARP4 significantly decreased in miR-424 mimic group in HGC-27 cells and increased in miR-424 inhibitor group in MKN-28 cells, compared with their NC group (Fig. 7c2). However, the luciferase activity of mutant circLARP4 had no difference between miR-424 group and NC group in these two cell lines. Moreover, the luciferase activity of wild type LATS1 3’UTR was decreased by miR-424 mimic but increased by co-transfection with miR-424 mimic and circLARP4 in HGC-27 cells (Additional file 5: Figure S4e), while the luciferase activity of wild type LATS1 3’UTR was increased by miR-424 inhibitor but decreased by co-transfection with miR-424 inhibitor and si-circLARP4 in MKN-28 cells (Additional file 5: Figure S4f). However, the luciferase activity of mutant LATS1 had no difference between NC, miR-424 and co-transfection groups (Additional file 5: Figure S4e, f).

Furthermore, we used the online circular RNA interactome to reveal a high degree of AGO2 occupancy in the region of circLARP4 (Additional file 1: Table S8). To confirm this result, we performed RNA immunoprecipitation (RIP) for AGO2 in HGC-27 and MKN-28 cells and investigated the expression level of endogenous circLARP4 and miR-424 pulled-down from AGO2-expressed cells by qRT-PCR analysis. The results indicated that Ago2 antibody precipitated the AGO2 protein from the cell lysates (Fig. 7d, up panel), and circLARP4 and miR-424 were highly expressed in the AGO2 pellet compared with those in the input control (Fig. 7d, down panel). We also observed the expression of LATS1, a target of miR-424 and its downstream gene YAP after co-transfection with circLARP4 and miR-424 mimic or si-circLARP4 and miR-424 inhibitor by qRT-PCR (Additional file 5: Figure S4d) and western blot analysis (Fig. 7e), indicating that circLARP4 revived LATS1 and p-YAP expression and decreased YAP expression and counteracted the effects of miR-424 on their expression in HGC-27 cells. Reversely, knockdown of circLARP4 reduced LATS1 and p-YAP expression and increased YAP expression and reversed the effect of miR-424 inhibitor on their expression in MKN-28 cells. Moreover, ectopic expression of circLARP4 attenuated the proliferative effect caused by miR-424 in HGC-27 cells, and knockdown of circLARP4 weakened the anti-proliferative effect induced by miR-424 inhibitor in MKN-28 cells (Fig. 7f). Altogether, these results inferred that circLARP4 could sponge miR-424 and inhibit its activity in GC cells.

### circLARP4 Acts as an independent prognostic factor for OS of GC patients

**Fig. 8:**
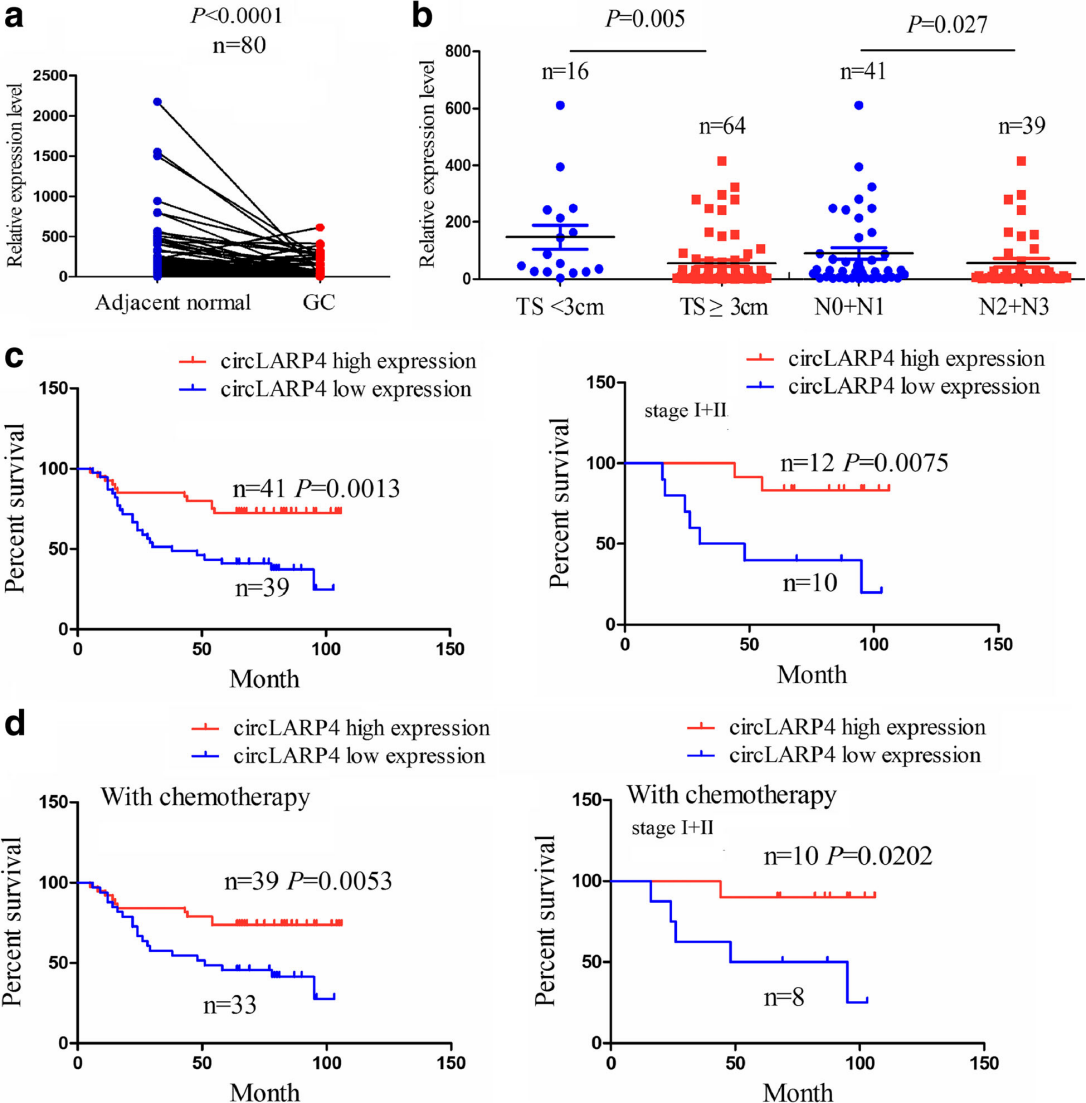
circLARP4 acts as an independent prognostic factor for OS of GC patients. **a** Schematic representation of the low expression level of circLARP4 in GC tissues compared with adjacent normal tissues by FISH analysis. **b** The expression levels of circLARP4 in GC patients with TS >3 cm or ≤3 cm and those with stage N2 + N3 or stage N0 + N1. **c** Kaplan-Meier analysis of the correlation of circLARP4 expression level with OS of GC patients or early stage ones (stageI + II). **d** Kaplan-Meier analysis of the correlation of circLARP4 expression level with therapeutic outcomes of GC patients or early stage ones (stageI + II) treated with adjuvant chemotherapy of oxaliplatin and 5-Fu

FISH analysis showed that the expression level of circLARP4 was markedly downregulated in GC tissues compared with the pair-matched normal tissues (Fig. 8a). In addition, we also found that circLARP4 expression level was decreased in GC patients with tumor size (TS) > 3 cm or stage N2 + N3 compared with those with TS ≤ 3 cm or stage N0 + N1 (Fig. 8b). We further analysed the correlation of circLARP4 expression level with various clinicopathological characteristics of 80 GC patients, who were divided into two groups-circLARP4 high expression and circLARP4 low expression according to the cut-off value (Additional file 6: Figure S5a). High expression of circLARP4 was found negatively associated with the tumor size and lymphatic metastasis, but had no correlation with other clinical and pathological parameters (Additional file 1: Table S9). Therefore, circLARP4 expression might be related with early stages of this disease. Kaplan-Meier analysis showed that GC patients or early stage patients (stageI + II) (log-rank test, Fig. 8c) rather than late stage ones (stage II + III or stage III) (log-rank test, Additional file 6: Figure S5b) with circLARP4 high expression had a significantly longer OS than those with circLARP4 low expression.

We then analysed the associations between circLARP4 expression level and the therapeutic outcomes in 72 GC patients treated with adjuvant chemotherapy of oxaliplatin and 5-Fu. These patients or early stage ones (Fig. 8d) rather than late stage ones (stage II + III or stage III) (Additional file 6: Figure S5c) with circLARP4 high expression had more favorable therapeutic outcome than those with circLARP4 low expression. As for the patients without chemotherapy, circLARP4 high or low expression had no impact on the survival of these patients (Additional file 6: Figure S5d).

To define whether the ability of circLARP4 to predict survival was independent of other clinicopathological factors of GC patients, univariate and multivariate Cox proportional hazards analyses revealed that circLARP4 level and lymphatic metastasis were independent prognostic factors for OS of GC patients (Additional file 1: Tables S10).

## Discussion

We have previously reported that LATS1 expression was downregulated in GC tissues [11]. Here, we verified the decreased expression of LATS1 in GC tissues by using the large sample size of TCGA sequencing data. Despite individual study having shown that miR-21 enhances radio-resistance of cervical cancer by targeting LATS1 [29], we here filtered out 5 miRNAs (miR-16, miR-15a, miR-15b, miR-590 and miR-424), which possessed very high stringency with LATS1 gene 3’UTR. The expressions levels of these miRNAs were upregulated in GC tissues, and except for miR-16, other miRNAs had negative correlation with LATS1 expression. Taken into account the strongest correlation of miR-424 with LATS1 expression in GC tissues, we analyzed the correlation of miR-424 and LATS1 expression with clinicopathological characteristic and prognosis of GC patients. We found that both of miR-424 high expression and LATS1 low expression were associated with pathological stage, OS and recurrence of GC patients, and miR-424 but not LATS1 gene was an independent prognostic factor for tumor recurrence of GC, suggesting a potential diagnostic marker for GC patients.

In view of the tissue diversity, aberrant expressions of miR-424 have been investigated in a variety of cancers. On the one hand, miR-424, downregulated in hepatocellular carcinoma (HCC) [30], cervical cancer [31] and esophageal carcinoma [32], inhibits cell growth and invasion [30–33], reverses epithelial-mesenchymal transition [34] and chemo-resistance [35], and strengthens the sensitivity of chemotherapy and radiotherapy [36, 37], indicating a tumor suppressive role in cancers. On the other hand, miR-424 promotes tumorigenesis and progression of prostate cancer [38] and reduces chemotherapy sensitivity by inhibiting apoptosis in breast cancer [39]. Our present studies showed that, miR-424 mimic stimulated cell growth and invasion, while miR-424 inhibitor reversed these effects by targeted regulation of LATS1 gene. Additionally, elevated miR-424 expression is also associated with metastasis and poor prognosis of non-small cell lung cancer [40] and accelerates gastric cancer proliferation [41]. These data support our findings that miR-424 may harbor an oncogenic role in GC.

Increasing evident shows that circRNAs are not simply by-products of splicing errors, rather they can modulate gene expression and act as miRNA sponge involved in cancer pathogenesis [25–27, 42]. CircRNAs can function as tumor suppressors or oncogenes in cancers. For example, circCCDC66, circ_0067934 and circHIAT1 promote tumor growth and metastasis [43–45], while circZKSCAN1 and circZNF292 suppress tumor progression by multiple signaling pathways [46, 47]. Here, we identified a circRNA derived from LARP4 gene locus, termed as circLARP4, which had the potential to sponge miR-424. Recent studies have shown that LARP4 as a La-related RNA-binding protein inhibits cancer cell migration and invasion [48]. We found that circLARP4 was differentially-expressed between GC and adjacent normal tissues, and was derived from Exon 9, 10 of the LARP4 gene and intermediate long intron. Compared with the linear LARP4, circLARP4 exhibited stable expression in GC cells in a time-dependent manner, and was mainly localized in the cytoplasm. Further functional experiments revealed that overexpression of circLARP4 inhibited DNA synthesis, cell proliferation and invasion by sponging miR-424 and regulating the expression of LATS1 and YAP genes, but knockdown of circLARP4 reversed these effects, suggesting that circLARP4 may function as a tumor suppressive factor in GC via regulation of miR-424/LATS1/YAP signaling pathway.

circRNAs can act as promising potential biomarkers for cancer diagnosis and prognosis due to their high stability and specific loop structure [43–45]. It has been reported that circPVT1, circ_0000190is and four-circRNA-based classifier are independent prognostic markers for survival and recurrence of patients with GC [49, 50, 51]. In this study, we also found that circLARP4 expression was downregulated in GC tissues, and was correlated with tumor size and lymphatic metastasis, and could act an independent prognostic marker for OS of GC patients as well as the patients with chemotherapy. Moreover, patients with circLARP4 high expression had a significantly better survival than those with circLARP4 low expression.

## Conclusion

**Fig. 9:**
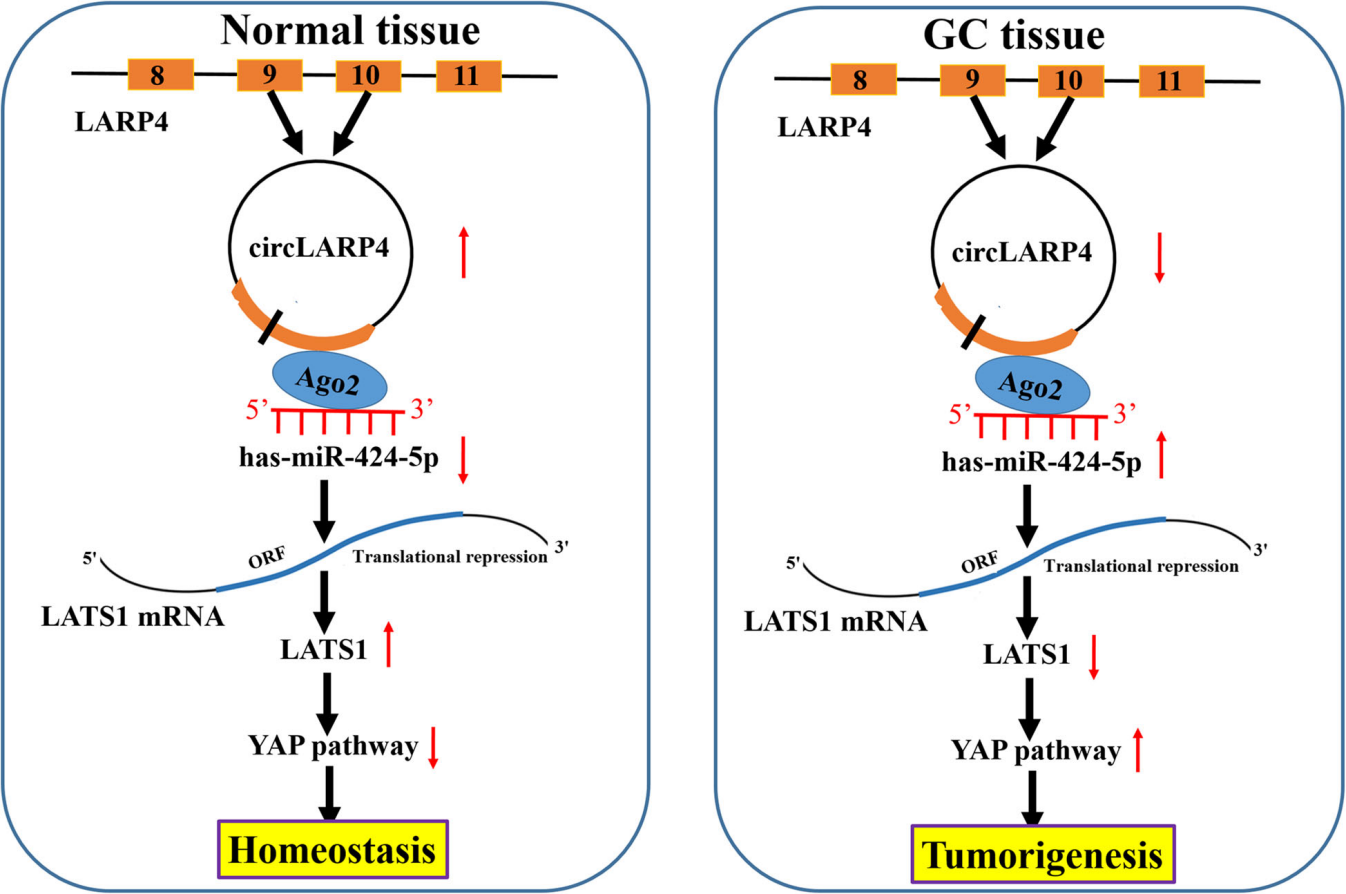
Schematic representation of the proposed mechanism of circLARP4 in GC cells. circLARP4 acted as a miR-424 sponge to regulate the miR-424/LATS1/YAP pathway in GC cells. Decreased circLARP4 expression in GC increased the activity of miR-424, which downregulated the expression of LATS1 and upregulated LATS1 downstream effector, thereby promoting gastric tumorigenesis and progression

In summary, we identified an oncogenic miR-424, which was negatively correlated with LATS1 expression. High expression of miR-424 or low expression of LATS1 was positively associated with pathological stage, OS and recurrence of patients with GC, and miR-424 promoted cell growth and invasion by targeting LATS1 gene. We further characterized a circLARP4 derived from LARP4 gene and demonstrated that circLARP4 was a tumor suppressive factor in GC by sponging miR-424 and regulating LATS1 expression (Fig. 9). The regulatory network involving circLARP4/miR-424/LATS1 axis may highlight a better understanding of gastric tumorigenesis and progression.

## Additional files

Additional file 1: Table S1. Clinicopathological data of GC patients from TCGA database. **Table S2** Clinicopathological data of GC patients from Tissue Microarray. **Table S3** List of primers of the genes. **Table S4** Correlation of LATS1 and miR-424 expression with clinicopathologic features of GC patients. **Table S5** Summary of univariate and multivariate Cox regression analysis of recurrence duration. **Table S6** Summary of univariate and multivariate Cox regression analysis of recurrence duration. **Table S7** Identification of circRNAs sponging miR-424 in gastric cancer. **Table S8** AGO2 binding sites in circLARP4 genomic region. **Table S9** Correlation of circLARP4 expression with clinicopathologic characteristics of GC patients. **Table S10** Summary of univariate and multivariate Cox regression analysis of overall survival duration. (DOCX 49 kb)

Additional file 2: Figure S1. The genetic alteration frequency and methylation levels of LATS1 in GC patients. **a** The genetic alteration frequency of LATS1 amplification, deletion and mutation in different pathological subtypes of GC. **b** The correlation of LATS1 gene expression with its putative copy number alterations in GC. **c** The correlation of LATS1 gene expression with its methylation level in GC. **d** The correlation of LATS1 gene expression with miR-15b-5p in GC. (PDF 2166 kb)

Additional file 3: Figure S2. The correlation of LATS1 and miR-424 expression with OS and recurrence of GC patients. **a and b** Kaplan Meier analysis of the correlation of LATS1 and miR-424 with OS of GC patients in TCTA RNA sequencing database. **c** Kaplan Meier analysis of the correlation of LATS1 expression with the recurrence of early stage patients (stage I + II) or late stage ones (stage III + IV). **d** Kaplan-Meier plotter analysis of the correlation of LATS1 expression with OS of GC patients with stage II or stage IV. (E) Kaplan-Meier plotter analysis of the correlation of LATS1 expression with recurrence of GC patients with stage II or stage III. (PDF 2418 kb)

Additional file 4: Figure S3. The effects of circLARP4 on GC cell proliferation. **a** The expression level of LATS1 was examined after transfection with miR-424 mimic and (or) LATS1 in HGC-27 cells, and miR-424 inhibitor and (or) sh-LATS1 in MKN-28 cells indicated by qRT-PCR. **b** The expression level of circLARP4 was detected in GC cell lines and GES-1 cells by qRT-PCR and spearman correlation analysis of the correlation of circLARP4 with miR-424 and LATS1 expression in GC cells. **c** Detection of cell proliferation of HGC-27 or MKN-28 cells transfected with circLARP4 overexpression or si-circLARP4 vectors by MTT assay. **d** Assessment of cell colony formation of HGC-27 or MKN-28 cells transfected with circLARP4 overexpression or si-circLARP4 vectors. *P < 0.05; **P < 0.01. (PDF 3665 kb)

Additional file 5: Figure S4. The binding sites of circLARP4 with miRNAs. **a** Schematic representation of potential binding sites of miRNAs with circLARP4. **b** The effects of miR-424 mimic or inhibitor on the expression level of circLARP4 in HCG-27 or MKN-28 cell line indicated by qRT-PCR. **c** The binding sites of wild type or mutant circLARP4 3’UTR with miR-424.-5p. **d** qRT-PCR analysis of the expression levels of LATS1 and YAP after transfection with circLARP4 + miR-424 in HGC-27 cells or si-circLARP4 + miR-424 inhibitor in MKN-28 cells. **e** the luciferase activity of wild type LATS1 3’UTR was examined by co-transfection with miR-424 mimic + circLARP4 in HGC-27 cells. **f** the luciferase activity of wild type LATS1 3’UTR was detected by co-transfection with miR-424 inhibitor + si-circLARP4 in MKN-28 cells. *P < 0.05; **P < 0.01. (PDF 2681 kb)

Additional file 6: Figure S5. Correlation of circLARP4 expression level with OS of GC patients. **a** Receiver operating characteristic (ROC) curve analysis of the cutoff value, sensitivity, specificity and AUC of circLARP4 in GC patients. **b** Kaplan-Meier analysis of the correlation of circLARP4 expression with OS of GC patients with stage II + III or stage III. **c** Kaplan-Meier analysis of the correlation of circLARP4 expression level with therapeutic outcomes of GC patients with stage II + III or stage III treated with adjuvant chemotherapy of oxaliplatin and 5-Fu. **d** Kaplan-Meier analysis of the correlation of circLARP4 expression level with therapeutic outcomes of GC patients without adjuvant chemotherapy. (PDF 3527 kb)
