# Supplementary material for: Circular RNA_LARP4 inhibits cell proliferation and invasion of gastric cancer by sponging miR-424-5p and regulating LATS1 expression
Source: Mol Cancer. 2017 Sep 11;16:151. doi: 10.1186/s12943-017-0719-3 (PMC5594516; doi:10.1186/s12943-017-0719-3)
Supplement: Supplementary file 1 — Clinicopathological data of GC patients from TCGA database. Table S2 Clinicopathological data of GC patients from Tissue Microarray. Table S3 List of primers of the genes. Table S4 Correlation of LATS1 and miR-424 expression with clinicopathologic features of GC patients. Table S5 Summary of univariate and multivariate Cox regression analysis of recurrence duration. Table S6 Summary of univariate and multivariate Cox regression analysis of recurrence duration. Table S7 Identification of circRNAs sponging miR-424 in gastric cancer. Table S8 AGO2 binding sites in circLARP4 genomic region. Table S9 Correlation of circLARP4 expression with clinicopathologic characteristics of GC patients. Table S10 Summary of univariate and multivariate Cox regression analysis of overall survival duration. (DOCX 49 kb) [file 12943_2017_719_MOESM1_ESM.docx]

Table S1 Clinicopathological data of GC patients from TCGA database

| Parameters | Cases n (%) |
| --- | --- |
| Total | 315 (100.00%) |
| ***Age*** |  |
| ≥60 | 211 (66.98%) |
| <60 | 104 (33.02%) |
| ***Gender*** |  |
| Female | 113 (35.87%) |
| Male | 202 (64.13%) |
| ***Tumor size (cm)*** |  |
| <2.5 | 275 (87.30%) |
| ≥2.5 | 40 (12.70%) |
| ***Pathological stage*** |  |
| I/II | 155 (49.21%) |
| III/IV | 160 (50.79%) |
| ***T classification*** |  |
| T1/T2 | 82 (26.03%) |
| T3/T4 | 233 (73.97%) |
| ***N classification*** |  |
| N0/N1 | 189 (60.00%) |
| N2/N3 | 126 (40.00%) |
| ***Distant metastasis*** |  |
| Negative | 298 (94.60%) |
| Positive | 17 (5.40%) |

Table S2 Clinicopathological data of GC patients from Tissue Microarray

| Parameters | Cases n (%) |
| --- | --- |
| Total | 80 (100.00%) |
| ***Age (y)*** |  |
| ≥60 | 46 (57.50%) |
| <60 | 34 (42.50%) |
| ***Gender*** |  |
| Male | 56 (70.00%) |
| Female | 24 (30.00%) |
| ***Tumor size (cm)*** |  |
| ≥3 | 64 (80.00%) |
| <3 | 16 (20.00%) |
| ***Pathological classification*** |  |
| Adenocarcinoma (AC) | 16 (20.00%) |
| Signet ring cell carcinoma (SRCC) | 59 (73.75%) |
| AC+SRCC | 5 (6.25%) |
| ***Pathological stage*** |  |
| Ⅰ+Ⅱ | 22 (27.50%) |
| Ⅱ+Ⅲ | 20 (25.00%) |
| Ⅲ | 38 (47.50%) |
| ***T classification*** |  |
| T1+T2 | 24 (30.00%) |
| T3+T4 | 56 (70.00%) |
| ***N classification*** |  |
| N0+N1 | 41 (51.25%) |
| N2+N3 | 39 (48.75%) |
| ***Distant metastasis*** |  |
| Negative | 75 (93.75%) |
| Positive | 5 (6.25%) |
| ***With chemotherapy*** |  |
| Negative | 8 (10.00%) |
| Positive | 72 (90.00%) |

Table S3 List of primers of the genes

| Genes | Forward primer | Reverse primer |
| --- | --- | --- |
| miR-424 | 5′-GGCTAGT CAGCAGCAATTCATGT-3′ | 5'-GTGCAGGGTCCGAGGT-3′ |
| LATS1 | 5′-GTTAAGGGGAGAGCCAGGTCCTT-3′ | 5′-TCAAGGAAGTCCCCAGG ACTGT-3′ |
| circLARP4 | 5′- GGGCATCAGGAGCAAACTTA -3′ | 5′-CTGGCGAATTAAAGCCATTC-3′ |
| YAP | 5′-TAGCCCTGCGTAGCCAGTTA-3′ | 5′- TCATGCTTAGTCCACTGTCTGT -3′ |
| GAPDH | 5’-AACTTTGGGATTGTGGAAGG-3’ | 5’-ACACA TTGGGGGTAGGAACA -3’ |
| U6 | 5′-GCTTCGGCAGCACATATACTAAAAT-3′ | 5′-CGCTTCACGAATTTGCGTGTCAT-3′ |

Table S4 Correlation of LATS1 and miR-424 expression with clinicopathologic features of GC patients

| clinicopathologic features | Cases  (n) | LATS1 | | *P* miR-424 | | | *P* |
| --- | --- | --- | --- | --- | --- | --- | --- |
|  | 315 | Low  207 | High  108 |  | Low  273 | High  42 |  |
| ***Age*** |  |  |  |  |  |  |  |
| ≥60 | 211 | 141 | 70 |  | 184 | 27 |  |
| <60 | 104 | 66 | 38 | 0.555 | 89 | 15 | 0.890 |
| ***Gender*** |  |  |  |  |  |  |  |
| Female | 113 | 75 | 38 |  | 96 | 17 |  |
| Male | 202 | 132 | 70 | 0.854 | 177 | 25 | 0.505 |
| ***Tumor size (cm)*** |  |  |  |  |  |  |  |
| <2.5 | 275 | 182 | 93 |  | 238 | 37 |  |
| ≥2.5 | 40 | 25 | 15 | 0.647 | 35 | 5 | 0.868 |
| ***Pathological stage*** |  |  |  |  |  |  |  |
| I/II | 155 | 114 | 41 |  | 128 | 27 |  |
| III/IV | 160 | 93 | 67 | 0.004 | 145 | 15 | 0.036 |
| ***T classification*** |  |  |  |  |  |  |  |
| T1/T2 | 82 | 57 | 25 |  | 67 | 15 |  |
| T3/T4 | 233 | 150 | 83 | 0.400 | 206 | 27 | 0.125 |
| ***N classification*** |  |  |  |  |  |  |  |
| N0/N1 | 189 | 131 | 58 |  | 160 | 29 |  |
| N2/N3 | 126 | 76 | 50 | 0.100 | 113 | 13 | 0.199 |
| ***Distant metastasis*** |  |  |  |  |  |  |  |
| Negative | 298 | 194 | 104 |  | 258 | 40 |  |
| Positive | 17 | 13 | 4 | 0.338 | 15 | 2 | 0.845 |

Table S5 Summary of univariate and multivariate Cox regression analysis of recurrence duration

| Parameter | Univariate analysis | | |  | Multivariate analysis | | |
| --- | --- | --- | --- | --- | --- | --- | --- |
|  | *P* | HR | 95%CI |  | *P* | HR | 95%CI |
| Age (≥60 vs. <60 years) | 0.889 | 0.966 | 0.594-1.571 |  | 0.840 | 0.951 | 0.585-1.546 |
| Gender (Male vs. Female) | 0.033 | 1.816 | 1.051-3.139 |  | 0.038 | 1.789 | 1.033-3.096 |
| Tumor size (≥3.5 vs. <3.5 cm) | 0.922 | 0.967 | 0.494-1.894 |  | NA |  |  |
| Pathological stage (I/II vs. III/IV) | 0.816 | 1.057 | 0.662-1.690 |  | NA |  |  |
| T classification (T1/T2 vs. T3/T4) | 0.259 | 0.747 | 0.454-1.228 |  | NA |  |  |
| N classification (N0/N1 vs. N2/N3) | 0.065 | 1.559 | 0.974-2.497 |  | NA |  |  |
| Distant metastasis (Positive vs. Negative) | 0.998 | 0.998 | 0.313-3.181 |  | NA |  |  |
| LATS1 expression (High vs. low) | 0.023 | 0.530 | 0.306-0.915 |  | 0.25 | 0.535 | 0.309-0.925 |

NA: not analyzed; NS: not significant.

Table S6 Summary of univariate and multivariate Cox regression analysis of recurrence duration

| Parameter | Univariate analysis | | |  | Multivariate analysis | | |
| --- | --- | --- | --- | --- | --- | --- | --- |
|  | *P* | HR | 95%CI |  | *P* | HR | 95%CI |
| Age (≥60 vs. <60 years) | 0.889 | 0.966 | 0.594-1.571 |  | 0.848 | 0.954 | 0.587-1.549 |
| Gender (Male vs. Female) | 0.033 | 1.816 | 1.051-3.139 |  | 0.016 | 1.965 | 1.134-3.407 |
| Tumor size (≥3.5 vs. <3.5 cm) | 0.922 | 0.967 | 0.494-1.894 |  | NA |  |  |
| Pathological stage (I/II vs. III/IV) | 0.816 | 1.057 | 0.662-1.690 |  | NA |  |  |
| T classification (T1/T2 vs. T3/T4) | 0.259 | 0.747 | 0.454-1.228 |  | NA |  |  |
| N classification (N0/N1 vs. N2/N3) | 0.065 | 1.559 | 0.974-2.497 |  | NA |  |  |
| Distant metastasis (Positive vs. Negative) | 0.998 | 0.998 | 0.313-3.181 |  | NA |  |  |
| miR-424 expression (High vs. low) | 0.001 | 2.434 | 1.421-4.169 |  | <0.0001 | 2.637 | 1.532-4.539 |

NA: not analyzed;

Table S7 Identification of circRNAs sponging miR-424 in gastric cancer

| circRNA | P-value | FDR | FC (abs) | Regulation | circRNA_type | chrom | strand | GeneSymbol |
| --- | --- | --- | --- | --- | --- | --- | --- | --- |
| hsa_circRNA_100038 | 0.000385563 | 0.029991744 | 1.9456285 | up | exonic | chr1 | - | RERE |
| hsa_circRNA_101057 | 0.000938565 | 0.033651473 | 2.9437863 | down | exonic | chr12 | + | LARP4 |
| hsa_circRNA_101781 | 0.001699314 | 0.038173093 | 2.1163653 | up | exonic | chr16 | - | XPO6 |
| hsa_circRNA_103310 | 0.002496748 | 0.040572148 | 2.1703245 | up | exonic | chr3 | + | RBMS3 |
| hsa_circRNA_102901 | 0.007212343 | 0.054745365 | 2.9300725 | up | exonic | chr2 | - | PLEKHM3 |
| hsa_circRNA_100519 | 0.008312348 | 0.057509389 | 2.2747457 | up | exonic | chr10 | - | LARP4B |
| hsa_circRNA_101805 | 0.008884671 | 0.058896588 | 2.4198067 | up | exonic | chr16 | + | PHKB |
| hsa_circRNA_101596 | 0.01136767 | 0.065170834 | 2.1597889 | down | exonic | chr15 | - | COX5A |
| hsa_circRNA_103106 | 0.011583444 | 0.065662777 | 8.1733342 | up | exonic | chr20 | + | DNAJC5 |
| hsa_circRNA_102934 | 0.013573043 | 0.069177325 | 1.5187419 | down | exonic | chr2 | + | AGFG1 |
| hsa_circRNA_103309 | 0.013833227 | 0.069642936 | 3.8207661 | up | exonic | chr3 | + | RBMS3 |
| hsa_circRNA_103472 | 0.01431632 | 0.069900057 | 2.9871368 | up | exonic | chr3 | - | TMCC1 |
| hsa_circRNA_103727 | 0.015238001 | 0.071885787 | 2.4710097 | down | exonic | chr4 | - | PDE5A |
| hsa_circRNA_100034 | 0.016763402 | 0.074729889 | 2.6748863 | down | exonic | chr1 | - | RERE |
| hsa_circRNA_000178 | 0.017462978 | 0.076300029 | 2.764266 | down | intronic | chr14 | + | SRSF5 |
| hsa_circRNA_100978 | 0.02125029 | 0.08466135 | 5.3512725 | up | exonic | chr11 | + | TBCEL |
| hsa_circRNA_102237 | 0.02131576 | 0.08466135 | 4.7289039 | down | exonic | chr17 | + | NARF |
| hsa_circRNA_102705 | 0.021854964 | 0.085845864 | 2.0643151 | down | exonic | chr2 | + | MTA3 |
| hsa_circRNA_104733 | 0.022394806 | 0.086432921 | 2.1605888 | up | exonic | chr9 | + | UHRF2 |
| hsa_circRNA_100037 | 0.023774232 | 0.088825772 | 1.7275842 | down | exonic | chr1 | - | RERE |
| hsa_circRNA_100033 | 0.023870618 | 0.088833841 | 3.8670585 | down | exonic | chr1 | - | RERE |
| hsa_circRNA_104976 | 0.024011065 | 0.088940284 | 1.8801502 | down | exonic | chrX | - | NLGN4X |
| hsa_circRNA_103357 | 0.026890659 | 0.093751784 | 3.4093857 | up | exonic | chr3 | - | SMARCC1 |
| hsa_circRNA_104881 | 0.03590171 | 0.109341217 | 1.5323314 | down | exonic | chr9 | + | HSDL2 |
| hsa_circRNA_100435 | 0.036350911 | 0.110143724 | 2.4875546 | down | exonic | chr1 | - | DSTYK |
| hsa_circRNA_103398 | 0.039148185 | 0.114323255 | 1.8215937 | down | exonic | chr3 | + | SLMAP |
| hsa_circRNA_103118 | 0.042211295 | 0.119326189 | 3.7106869 | up | exonic | chr21 | + | BACH1 |
| hsa_circRNA_101270 | 0.042489186 | 0.119535242 | 7.5459408 | up | exonic | chr13 | + | TDRD3 |
| hsa_circRNA_103116 | 0.048208531 | 0.128583182 | 2.2604328 | up | exonic | chr21 | + | BACH1 |
| hsa_circRNA_103810 | 0.049438815 | 0.130109361 | 1.5431091 | down | exonic | chr5 | - | ZFR |

Table S8 AGO2 binding sites in circLARP4 genomic region

| circRNA | Tag Name | % Identity | Alignment Length | Mismatches | Gap Openings | Tag Start | Tag End | circRNA Start | circRNA ENd | Upstream/  Downstream |
| --- | --- | --- | --- | --- | --- | --- | --- | --- | --- | --- |
| circLARP4 | HHFCT_37334_cluster-4914_3_27_31 | 100.00 | 31 | 0 | 0 | 1 | 31 | -732 | -702 | Upstream |
| circLARP4 | HPCB1_8868_G11142.1_50847450_32 | 100.00 | 32 | 0 | 0 | 1 | 32 | -678 | -647 | Upstream |
| circLARP4 | HPCB3_4799_G6621.1_50847320_26 | 100.00 | 26 | 0 | 0 | 1 | 26 | -784 | -759 | Upstream |
| circLARP4 | HPRT3_5895_G7746.1_50847321_25 | 100.00 | 25 | 0 | 0 | 1 | 25 | -792 | -768 | Upstream |
| circLARP4 | HPSEF_5036_G6015.1_50847357_38 | 100.00 | 38 | 0 | 0 | 1 | 38 | -755 | -718 | Upstream |
| circLARP4 | HPSEF_7668_G9214.1_36910204_32 | 100.00 | 32 | 0 | 0 | 1 | 32 | -755 | -724 | Upstream |
| circLARP4 | HPSLB_1690_G2053.1_50847384_36 | 100.00 | 36 | 0 | 0 | 1 | 36 | -731 | -696 | Upstream |

Table S9 Correlation of circLARP4 expression with clinicopathologic characteristics of GC patients

| Variables | Cases  (n) | circLARP4 | | *P* value |
| --- | --- | --- | --- | --- |
|  |  | High | Low |  |
| Total | 80 | 41 | 39 |  |
| ***Age (y)*** |  |  |  |  |
| ≥60 | 46 | 25 | 21 |  |
| <60 | 34 | 16 | 18 | 0.522 |
| ***Gender*** |  |  |  |  |
| Male | 56 | 30 | 26 |  |
| Female | 24 | 11 | 13 | 0.528 |
| ***Tumor size (cm)*** |  |  |  |  |
| ≥3 | 64 | 26 | 38 |  |
| <3 | 16 | 15 | 1 | <0.001 |
| ***Pathological classification*** |  |  |  |  |
| Adenocarcinoma (AC) | 16 | 7 | 9 |  |
| Signet ring cell carcinoma (SRCC) | 59 | 32 | 27 | 0.666 |
| AC+SRCC | 5 | 2 | 3 |  |
| ***Pathological stage*** |  |  |  |  |
| Ⅰ+Ⅱ | 22 | 12 | 10 |  |
| Ⅱ+Ⅲ | 20 | 14 | 6 | 0.084 |
| Ⅲ | 38 | 15 | 23 |  |
| ***T classification*** |  |  |  |  |
| T1+T2 | 24 | 16 | 8 |  |
| T3+T4 | 56 | 25 | 31 | 0.073 |
| ***N classification*** |  |  |  |  |
| N0+N1 | 41 | 26 | 15 |  |
| N2+N3 | 39 | 15 | 24 | 0.027 |
| ***Distant metastasis*** |  |  |  |  |
| Negative | 75 | 40 | 35 |  |
| Positive | 5 | 1 | 4 | 0.151 |
| ***With chemotherapy*** |  |  |  |  |
| Negative | 8 | 2 | 6 |  |
| Positive | 72 | 39 | 33 | 0.120 |

Table S10 Summary of univariate and multivariate Cox regression analysis of overall survival duration

| Parameter | Univariate analysis | | |  | Multivariate analysis | | |
| --- | --- | --- | --- | --- | --- | --- | --- |
|  | *P* | HR | 95%CI |  | *P* | HR | 95%CI |
| Age (≥60 vs. <60 years) | 0.670 | 1.153 | 0.598-2.224 |  | NA |  |  |
| Gender (Male vs. Female) | 0.993 | 1.003 | 0.495-2.032 |  | NA |  |  |
| Tumor size (≥3 vs. <3 cm) | 0.021 | 5.388 | 1.294-22.435 |  | 0.436 | 1.868 | 0.388-8.995 |
| Pathological classification (AC vs. SRCC vs. AC+SRCC) | 0.052 | 1.597 | 0.996-2.561 |  | NA |  |  |
| Pathological stage (III vs II+ III vs. Ⅰ+Ⅱ) | 0.388 | 1.182 | 0.808-1.731 |  | NA |  |  |
| T classification (T3+T4 vs. T1+T2) | 0.149 | 1.781 | 0.813-3.999 |  | NA |  |  |
| N classification (N2+N3 vs. N0+N1) | 0.000 | 4.421 | 2.122-9.208 |  | 0.003 | 3.237 | 1.473-7.114 |
| Distant metastasis (Positive vs. Negative) | 0.003 | 4.460 | 1.672-11.895 |  | 0.105 | 2.297 | 0.840-6.284 |
| With chemotherapy (Positive vs. Negative) | 0.055 | 0.421 | 0.174-1.017 |  | NA |  |  |
| circLARP4 expression (High vs. low) | 0.002 | 0.363 | 0.181-0.726 |  | 0.036 | 0.502 | 0.240-1.048 |

NA: not analyzed;
