# Supplementary figures and images for: Circular RNA_LARP4 inhibits cell proliferation and invasion of gastric cancer by sponging miR-424-5p and regulating LATS1 expression
Source: Mol Cancer. 2017 Sep 11;16:151. doi: 10.1186/s12943-017-0719-3 (PMC5594516; doi:10.1186/s12943-017-0719-3)

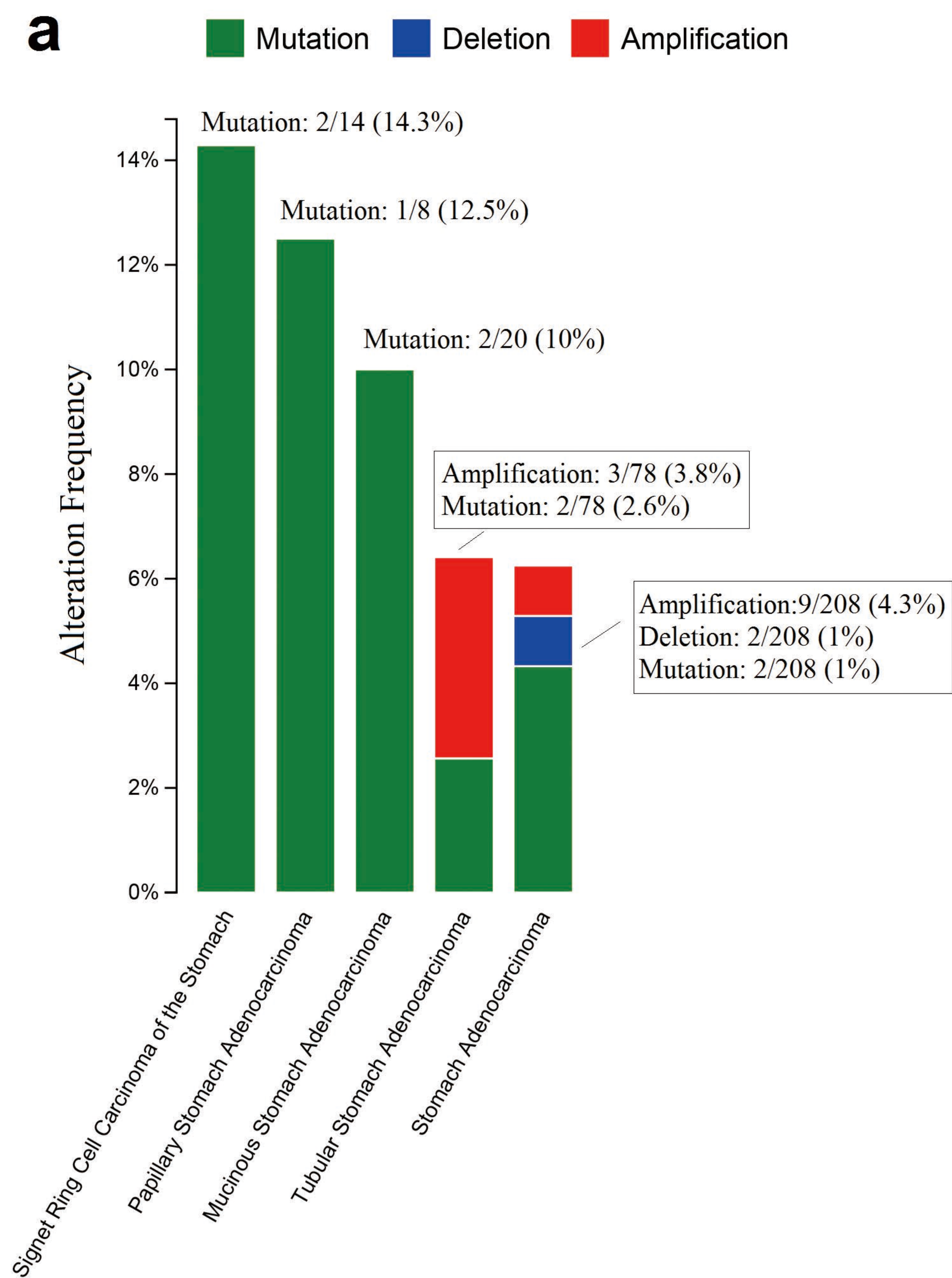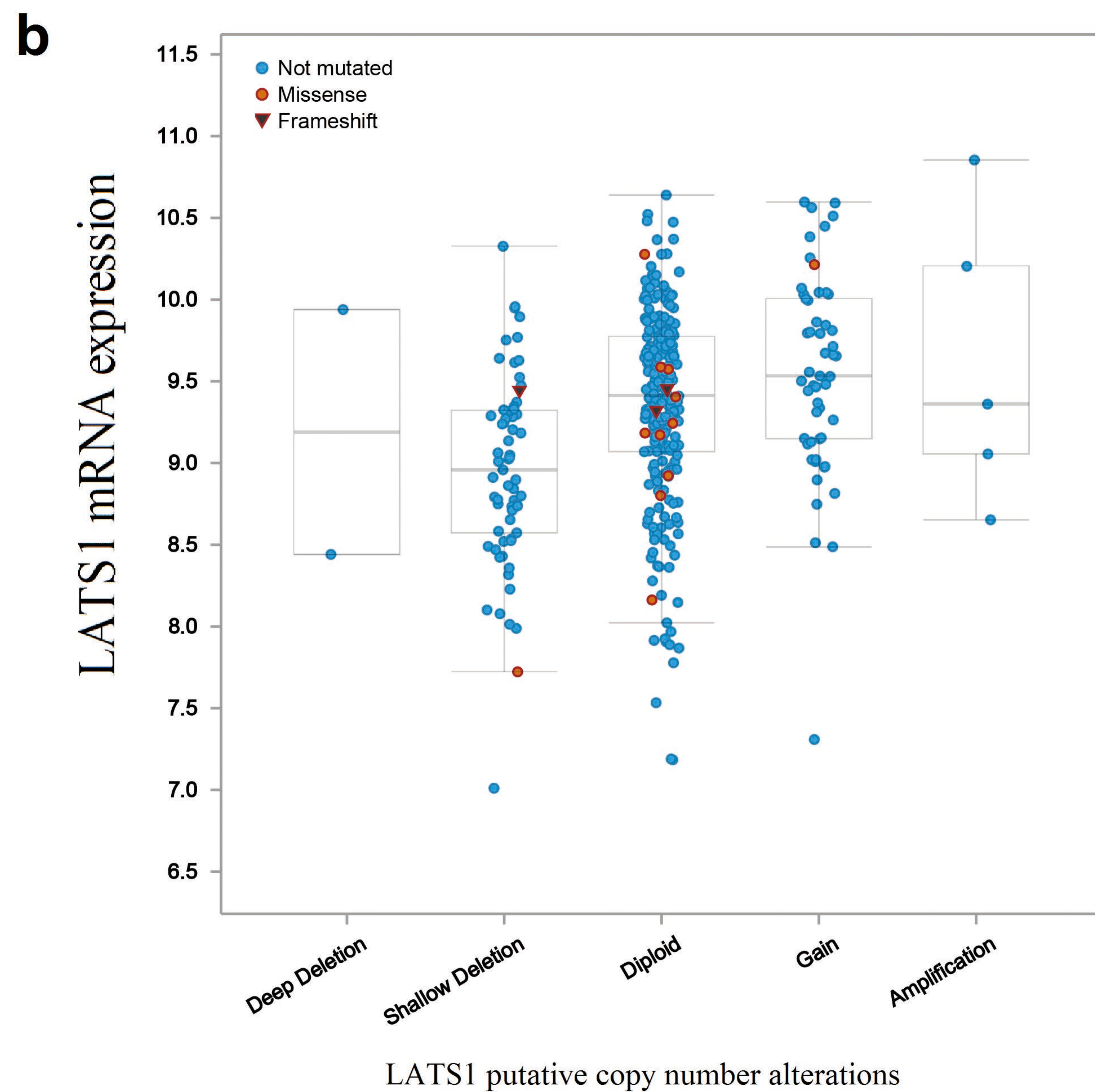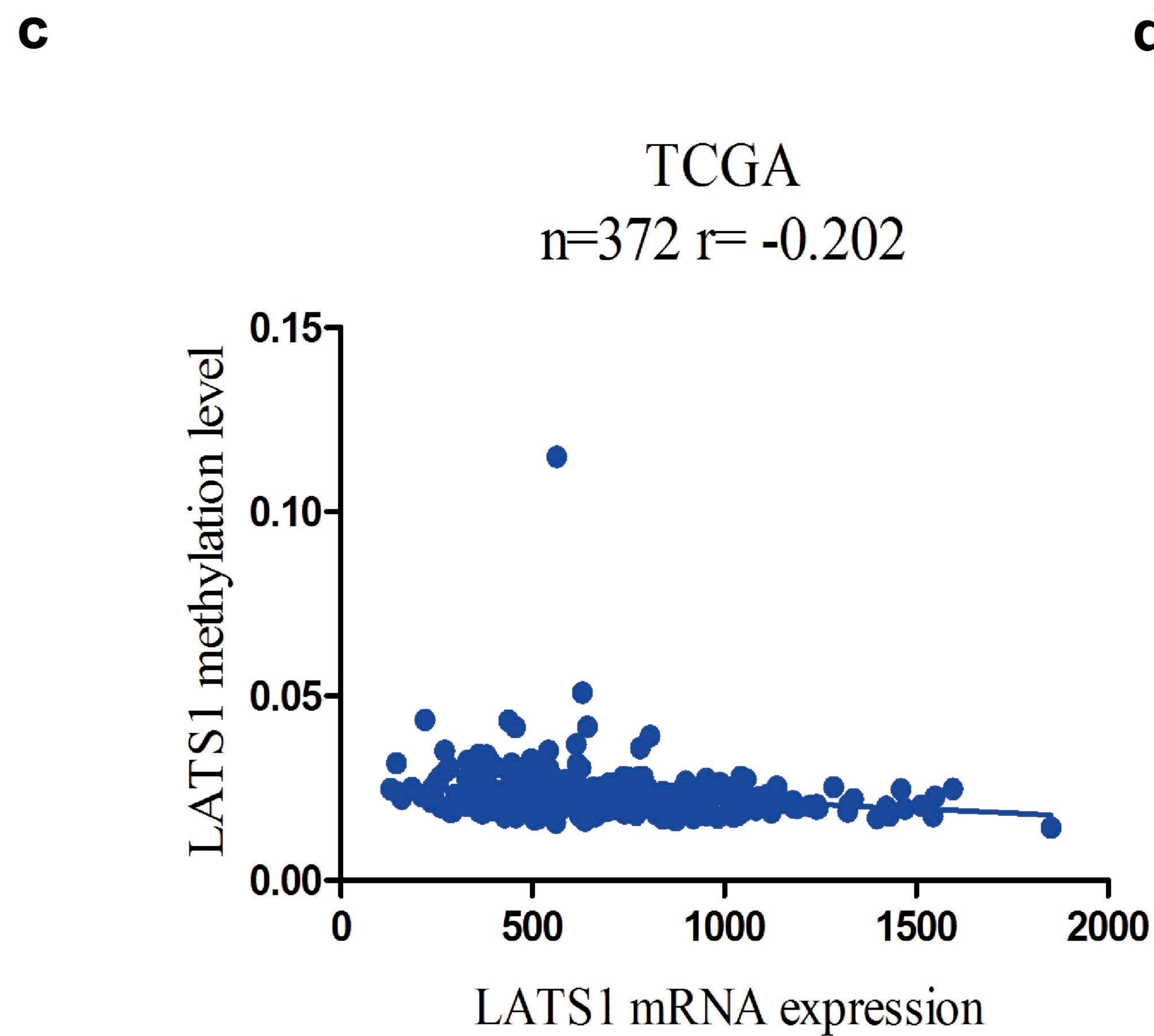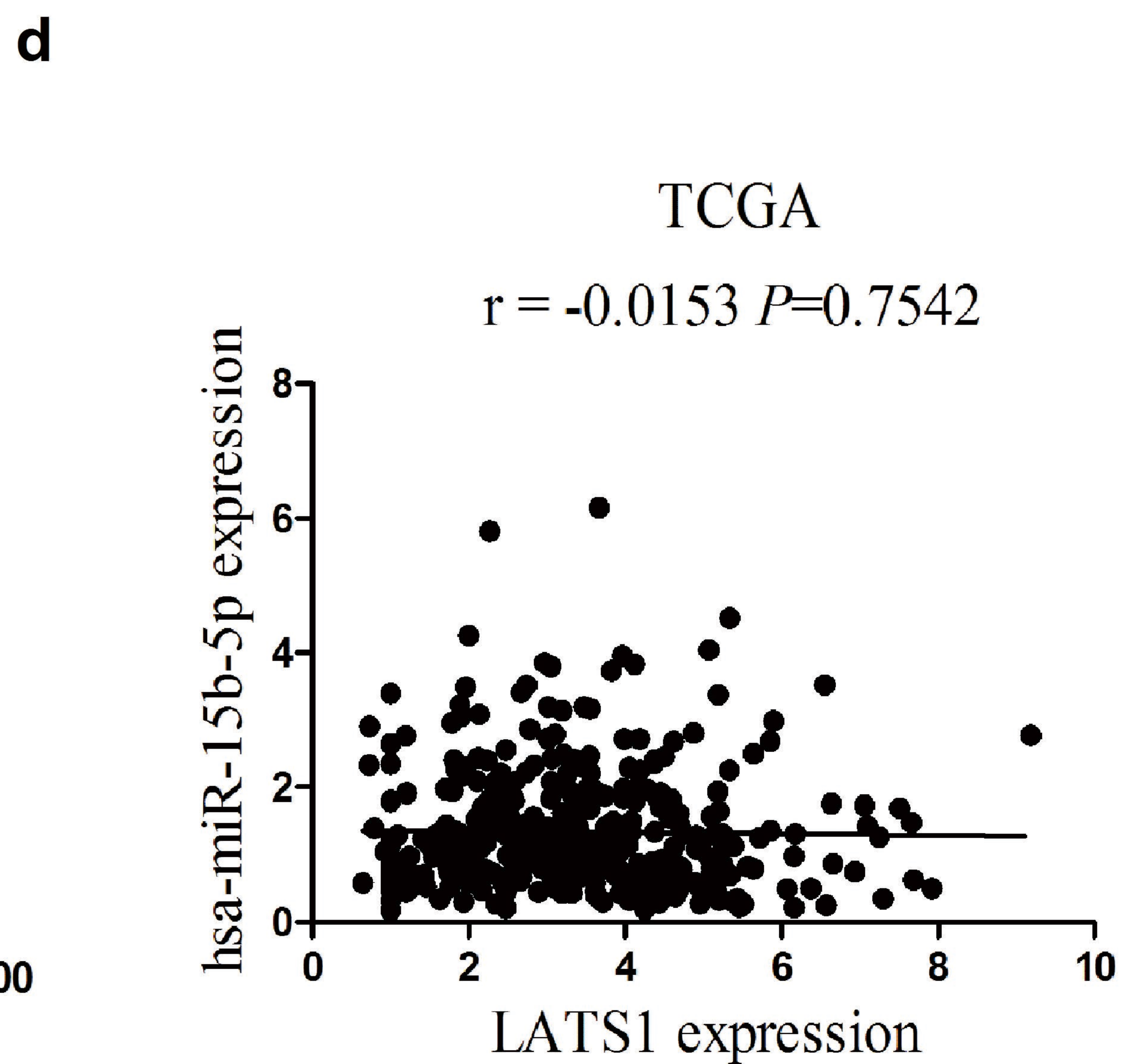

Supplement: Supplementary file 2 — The genetic alteration frequency and methylation levels of LATS1 in GC patients. a The genetic alteration frequency of LATS1 amplification, deletion and mutation in different pathological subtypes of GC. b The correlation of LATS1 gene expression with its putative copy number alterations in GC. c The correlation of LATS1 gene expression with its methylation level in GC. d The correlation of LATS1 gene expression with miR-15b-5p in GC. (PDF 2166 kb) [file 12943_2017_719_MOESM2_ESM.pdf]

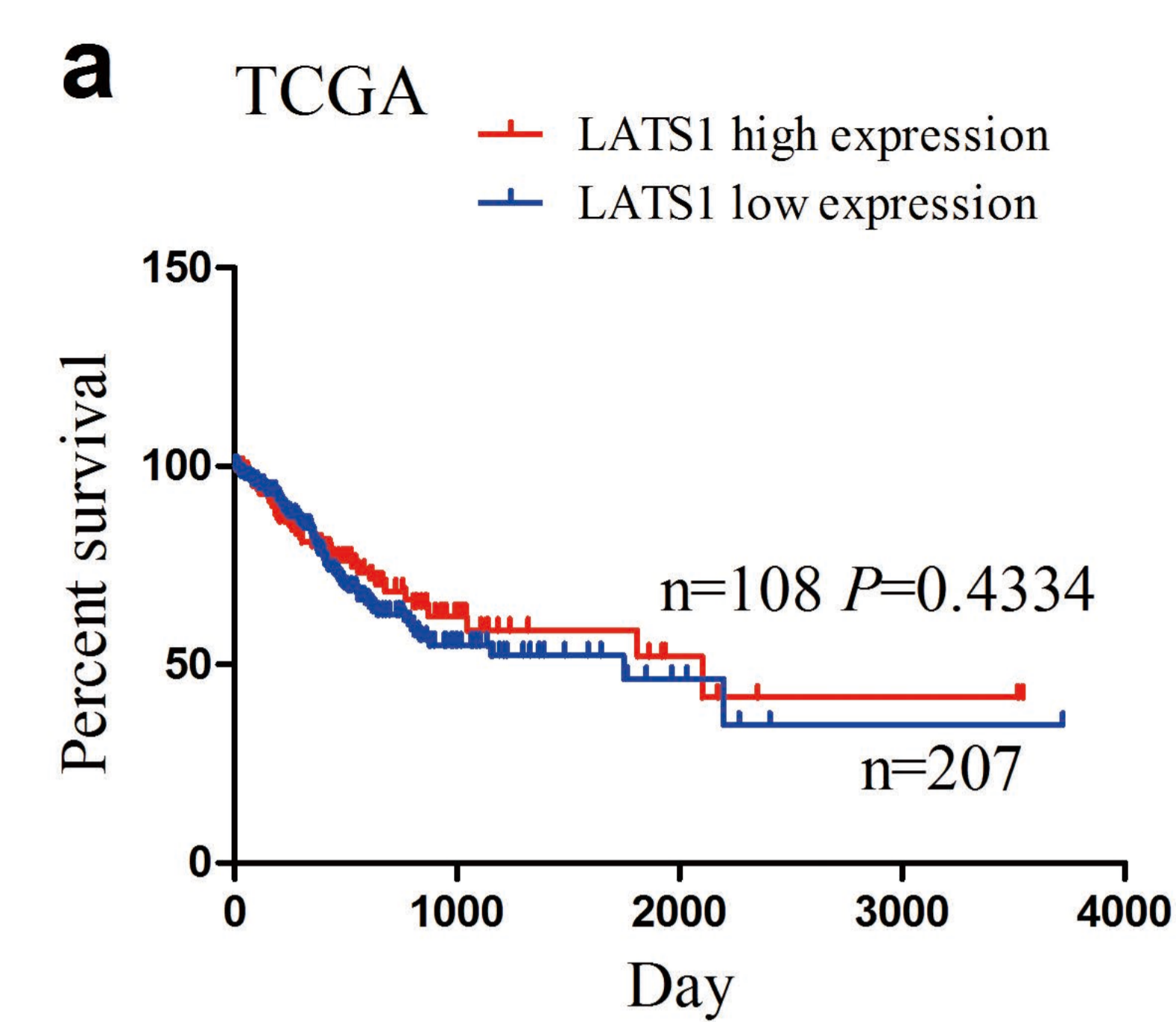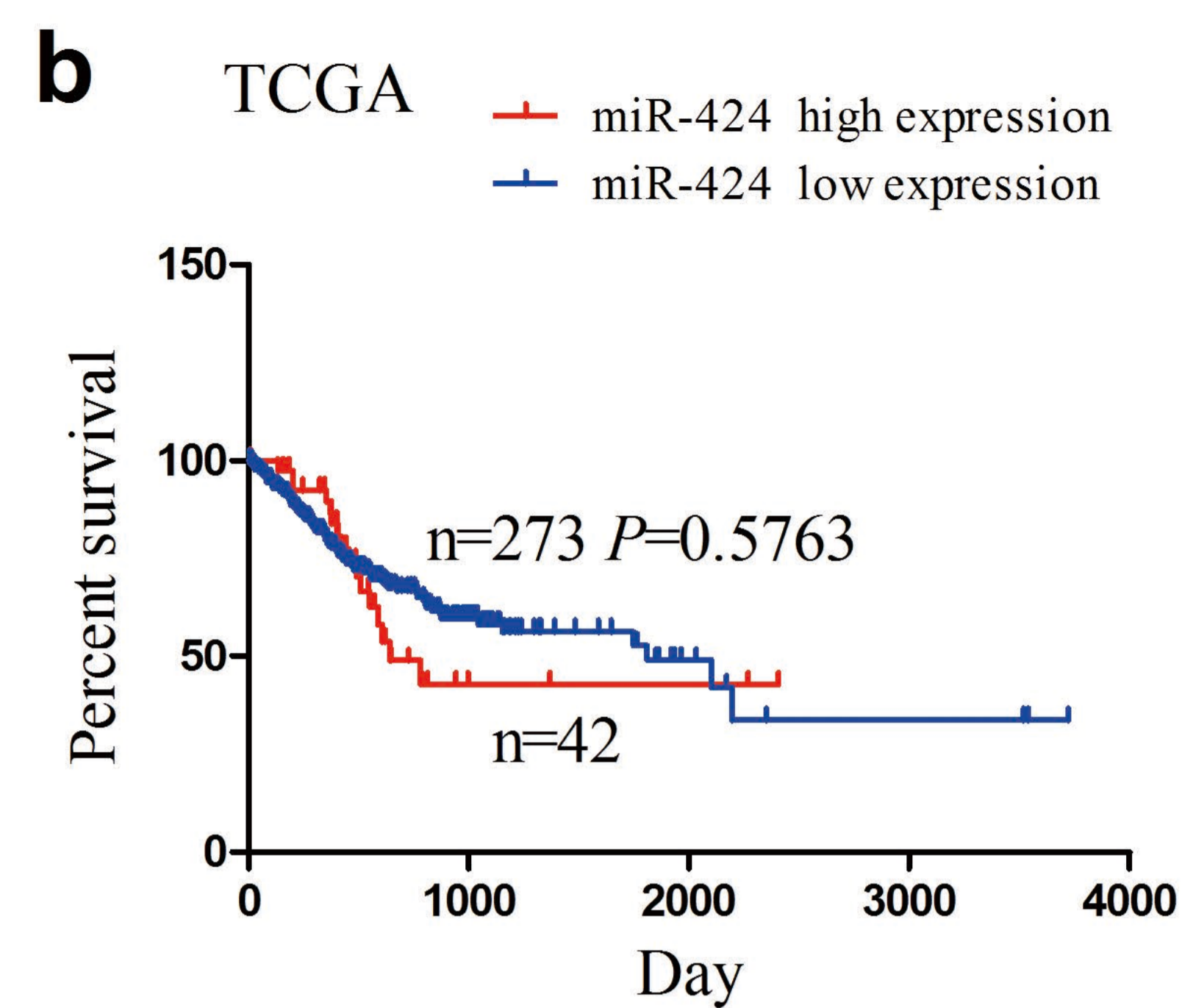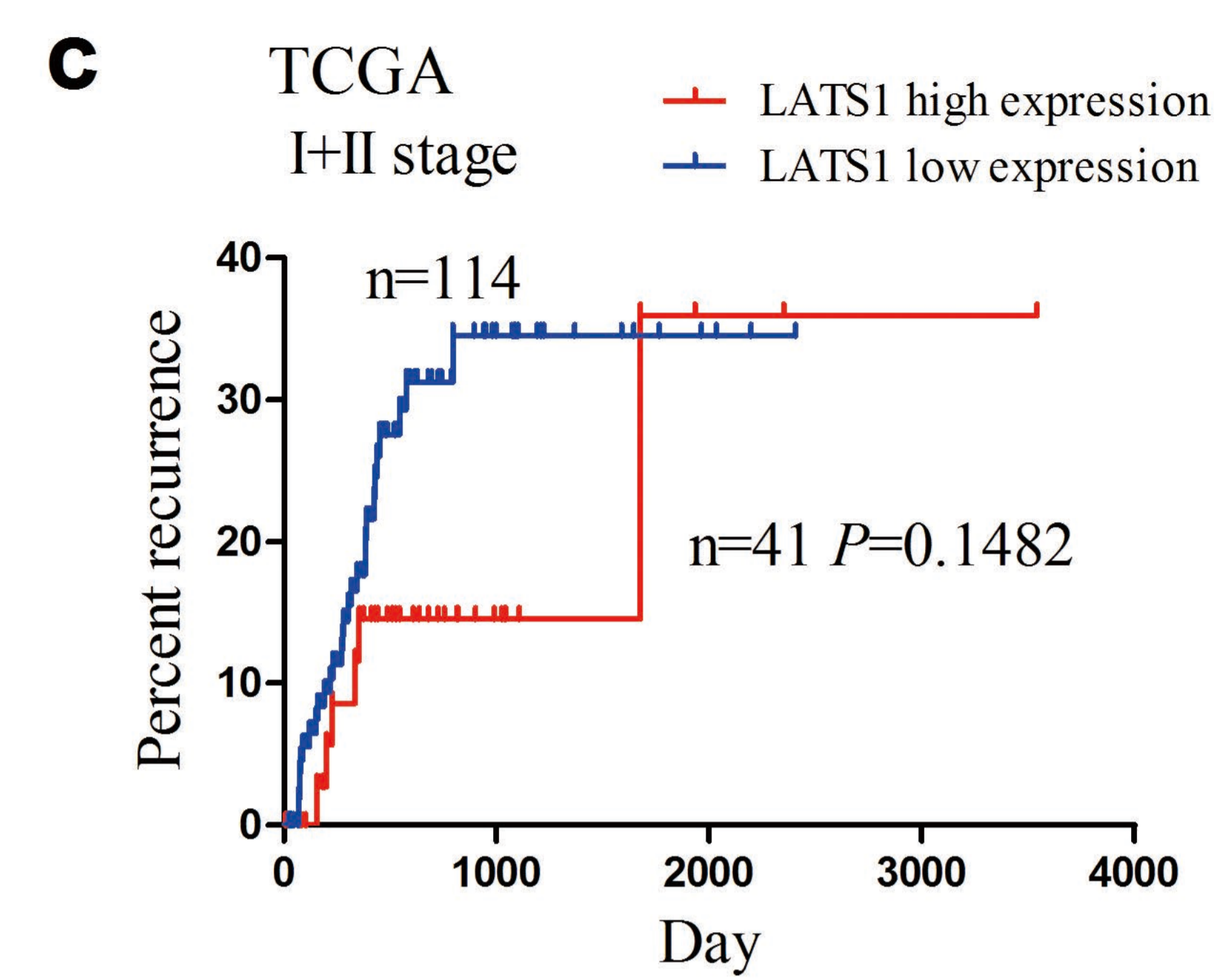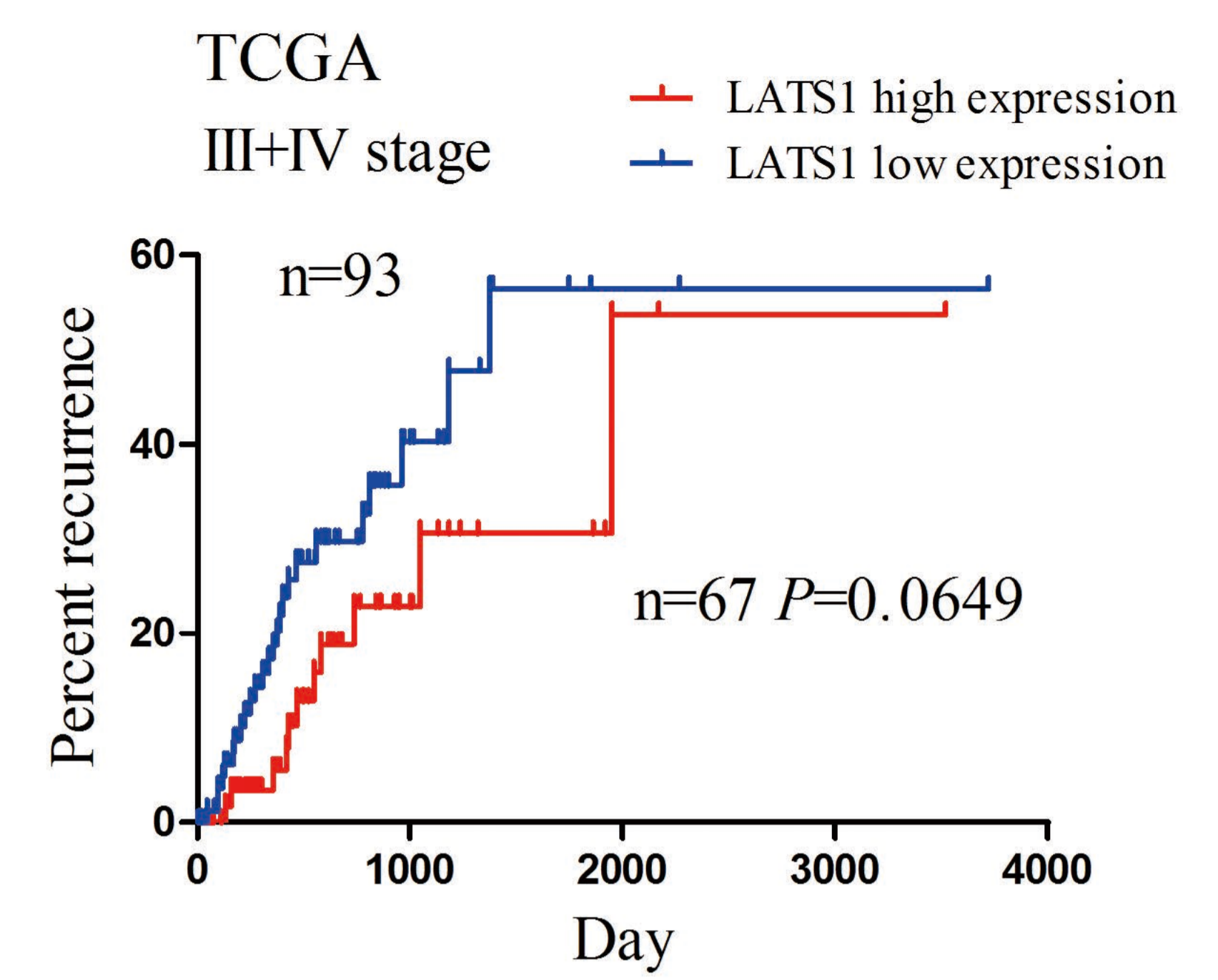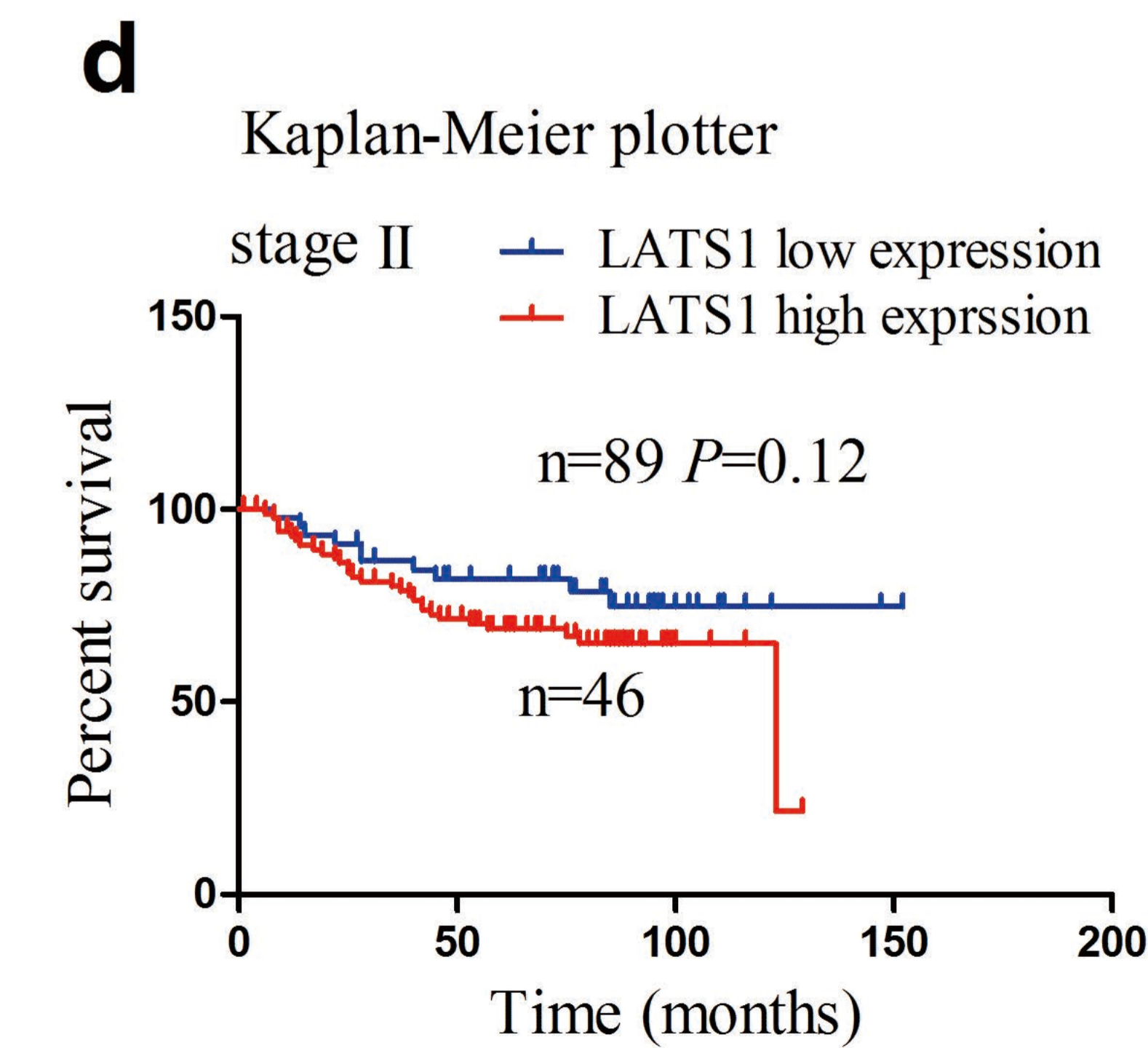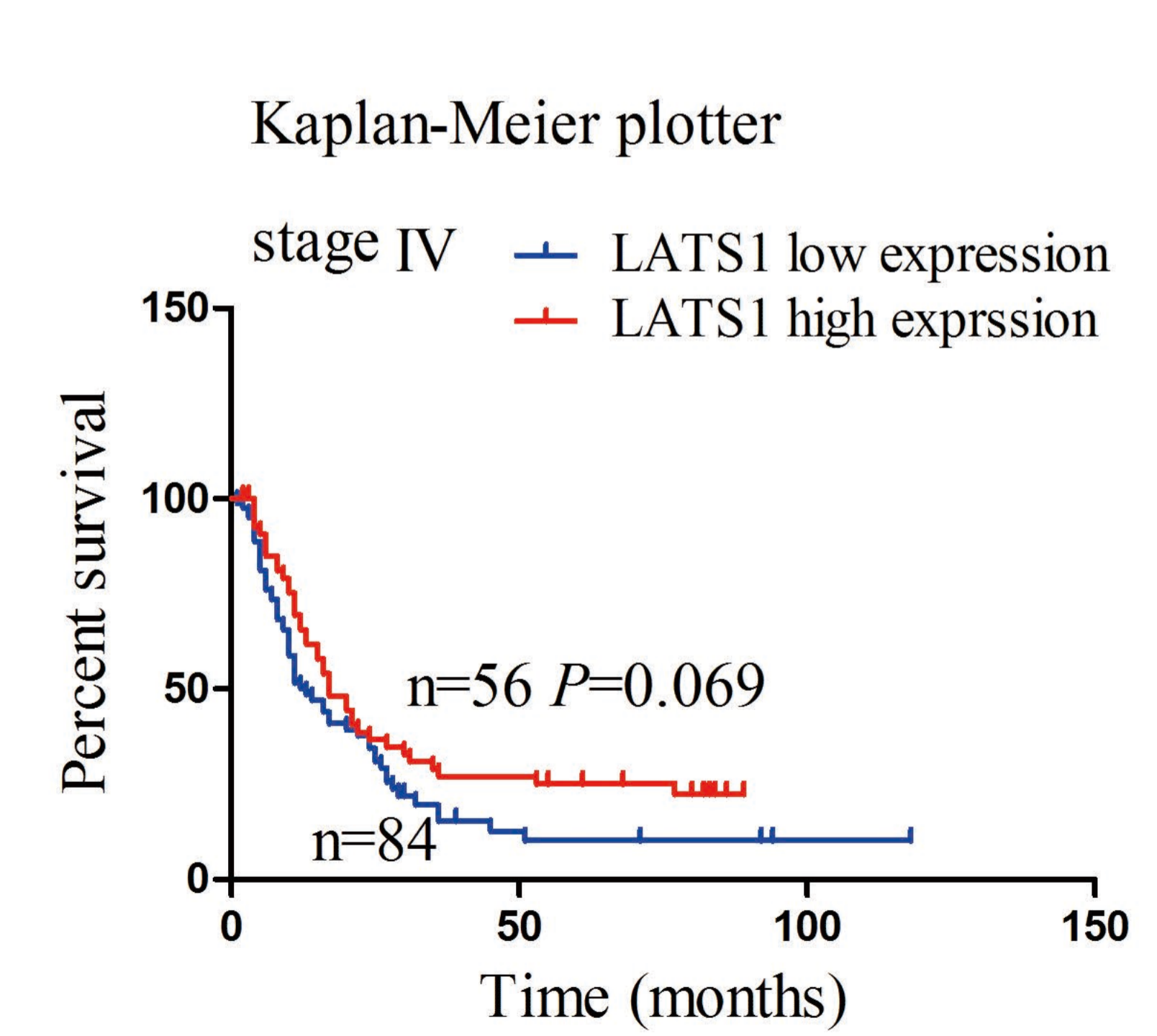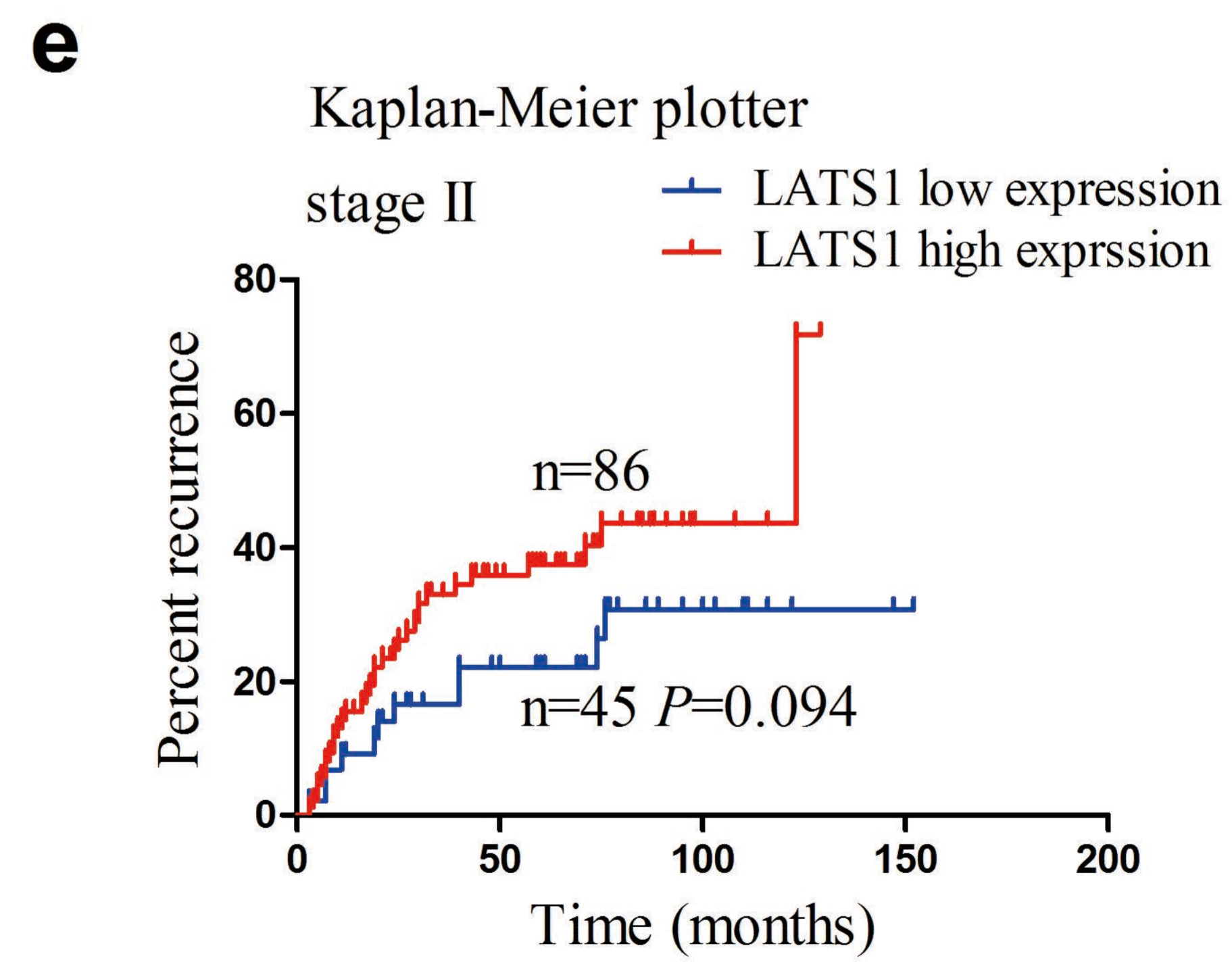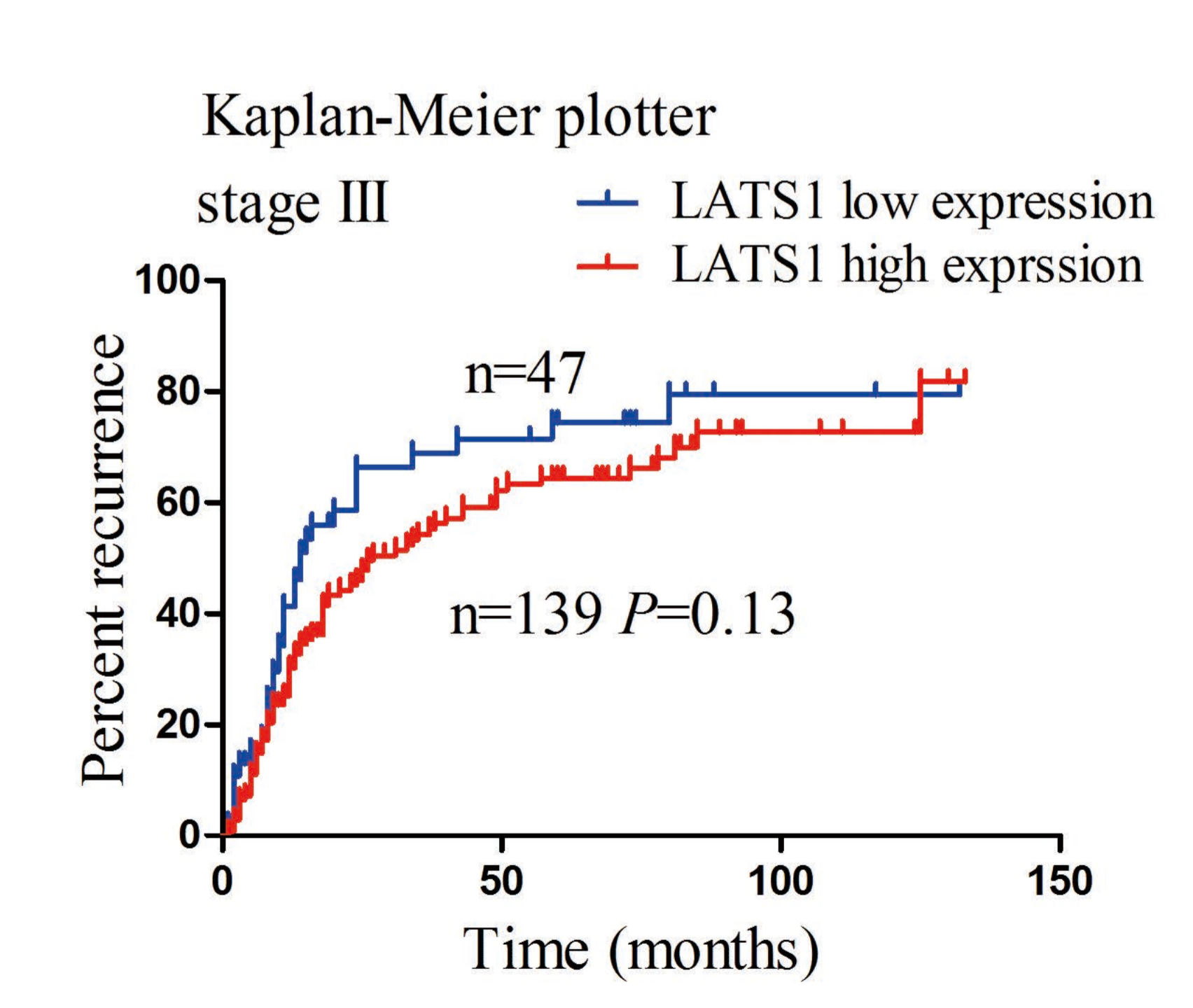

Supplement: Supplementary file 3 — The correlation of LATS1 and miR-424 expression with OS and recurrence of GC patients. a and b Kaplan Meier analysis of the correlation of LATS1 and miR-424 with OS of GC patients in TCTA RNA sequencing database. c Kaplan Meier analysis of the correlation of LATS1 expression with the recurrence of early stage patients (stage I + II) or late stage ones (stage III + IV). d Kaplan-Meier plotter analysis of the correlation of LATS1 expression with OS of GC patients with stage II or stage IV. (E) Kaplan-Meier plotter analysis of the correlation of LATS1 expression with recurrence of GC patients with stage II or stage III. (PDF 2418 kb) [file 12943_2017_719_MOESM3_ESM.pdf]

**a**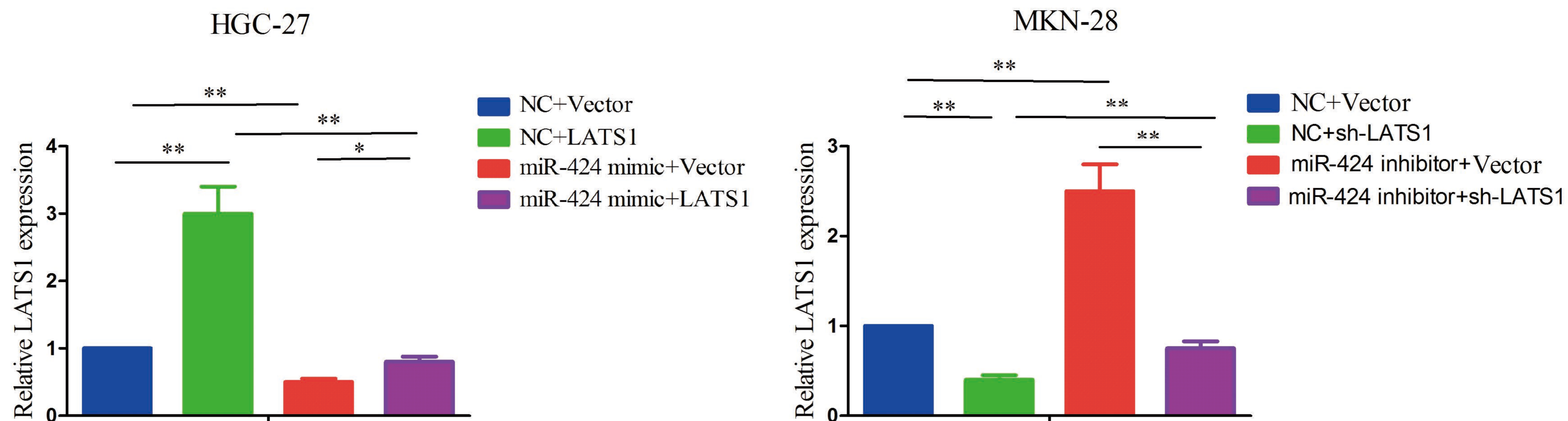**b**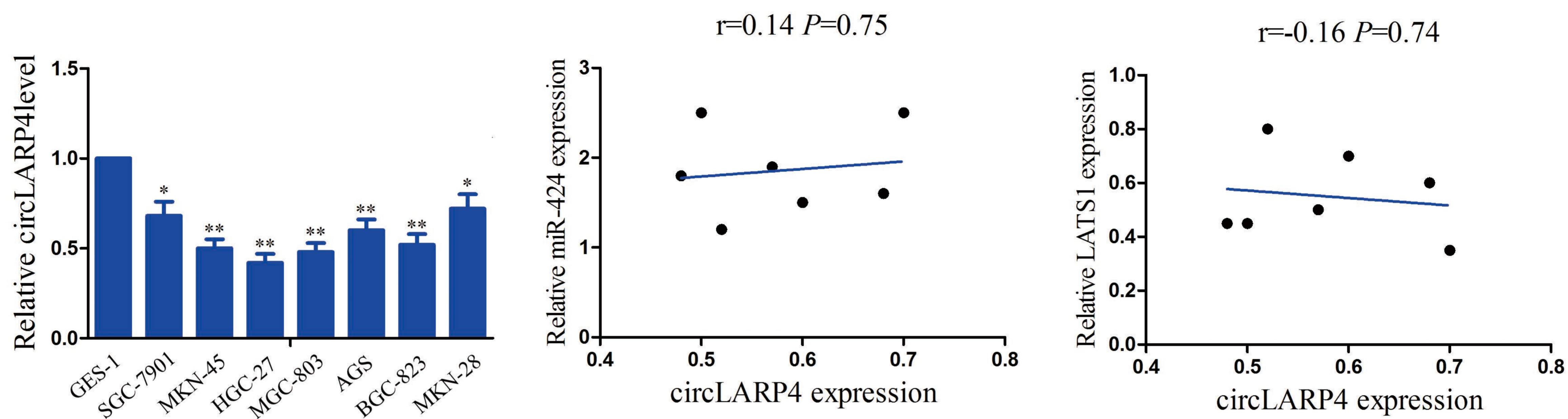**c**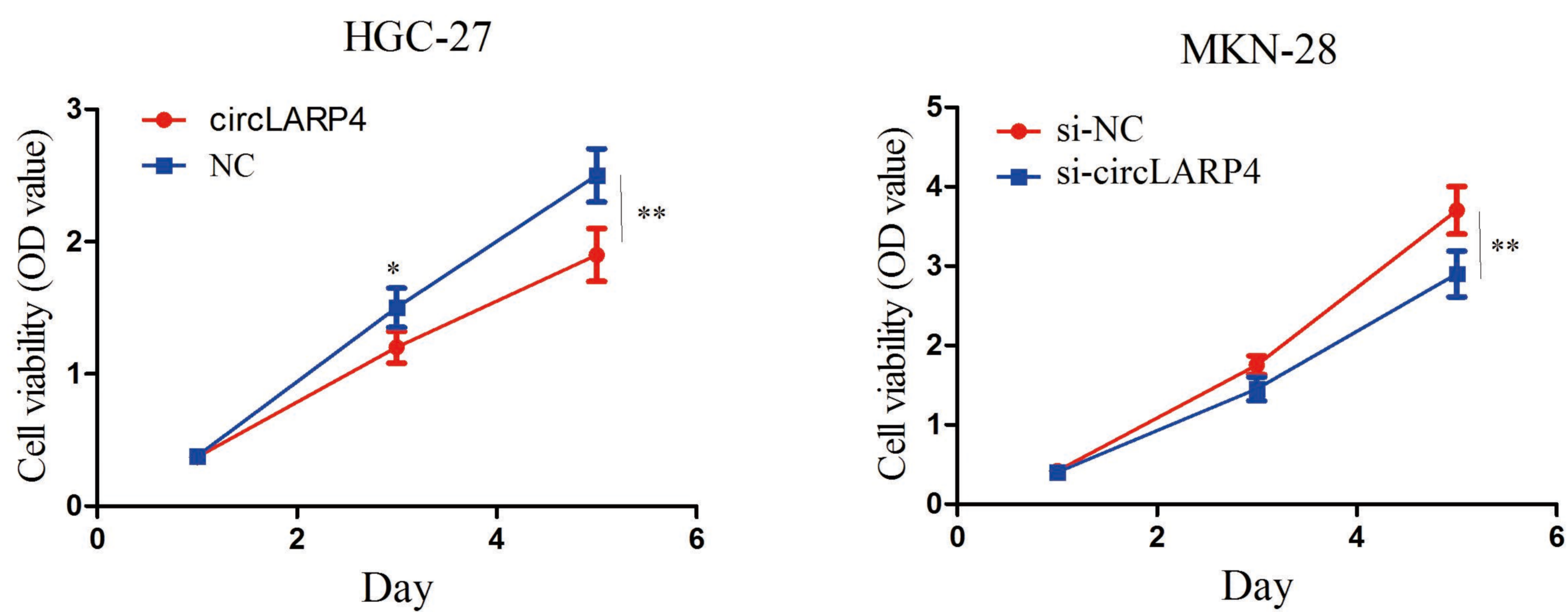**d**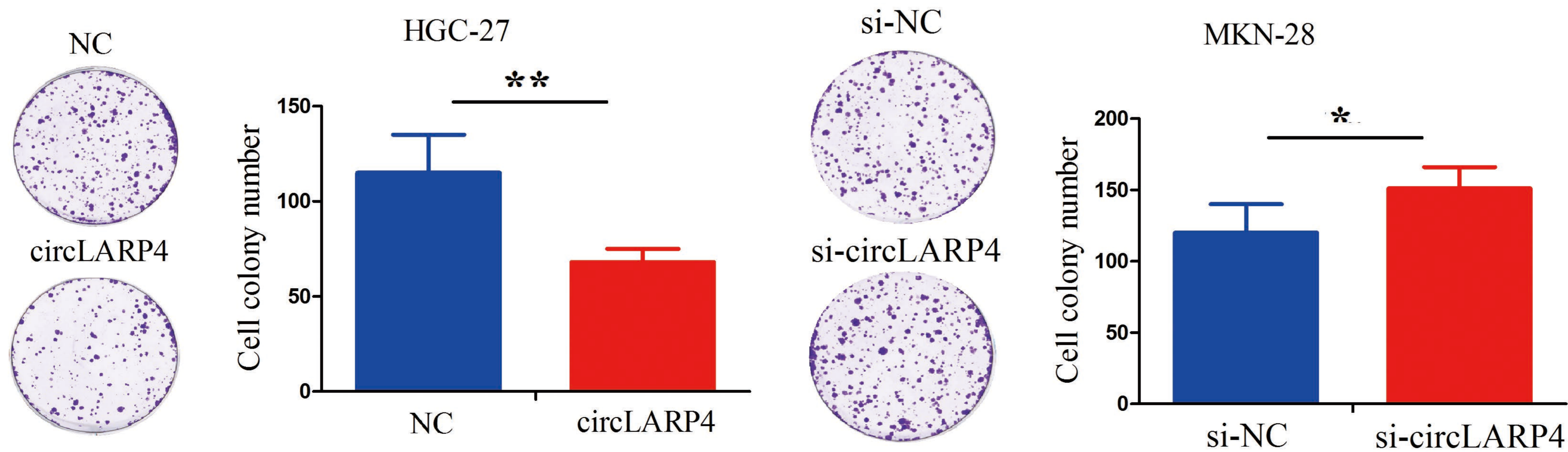

Supplement: Supplementary file 4 — The effects of circLARP4 on GC cell proliferation. a The expression level of LATS1 was examined after transfection with miR-424 mimic and (or) LATS1 in HGC-27 cells, and miR-424 inhibitor and (or) sh-LATS1 in MKN-28 cells indicated by qRT-PCR. b The expression level of circLARP4 was detected in GC cell lines and GES-1 cells by qRT-PCR and spearman correlation analysis of the correlation of circLARP4 with miR-424 and LATS1 expression in GC cells. c Detection of cell proliferation of HGC-27 or MKN-28 cells transfected with circLARP4 overexpression or si-circLARP4 vectors by MTT assay. d Assessment of cell colony formation of HGC-27 or MKN-28 cells transfected with circLARP4 overexpression or si-circLARP4 vectors. *P < 0.05; **P < 0.01. (PDF 3665 kb) [file 12943_2017_719_MOESM4_ESM.pdf]

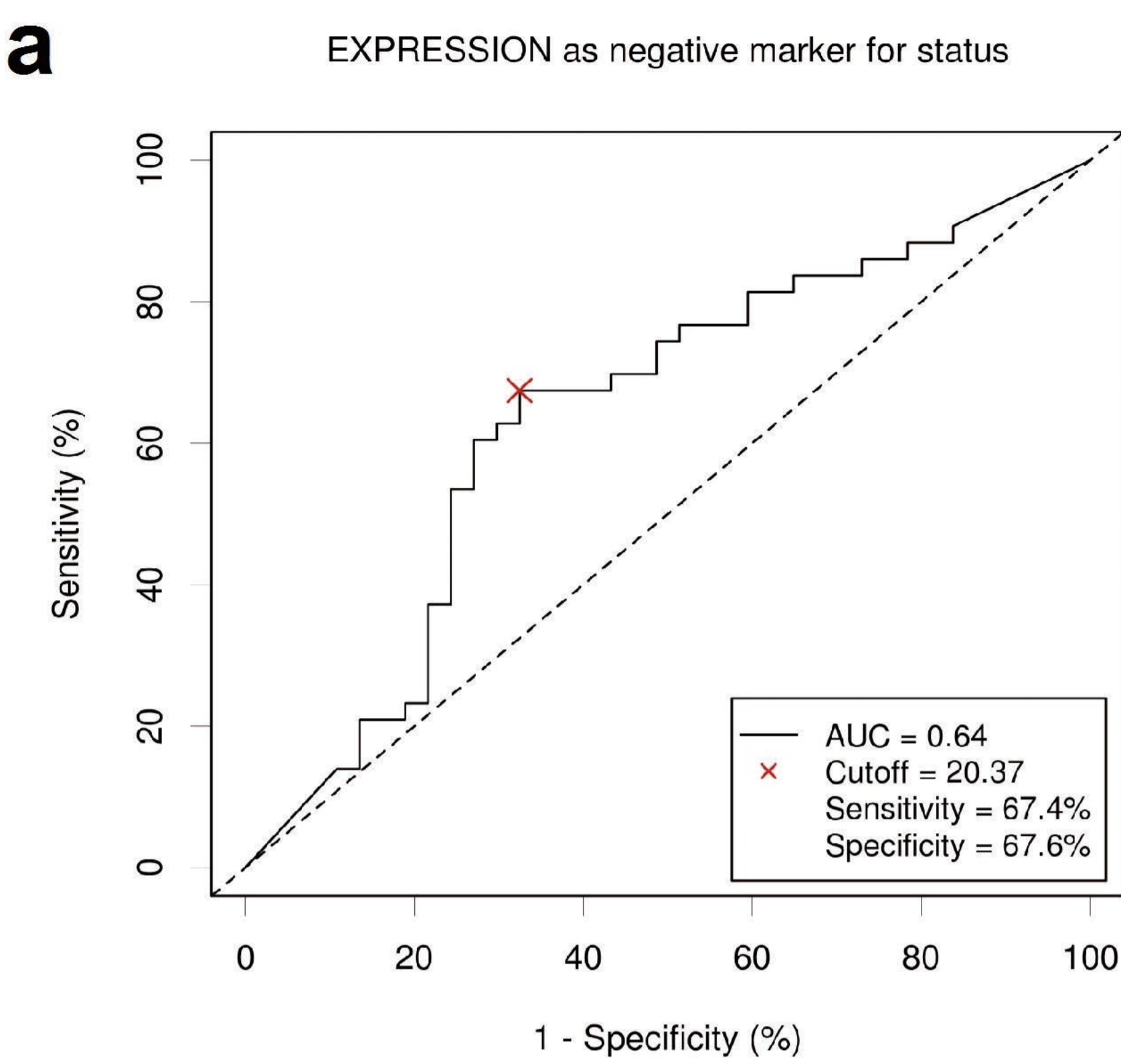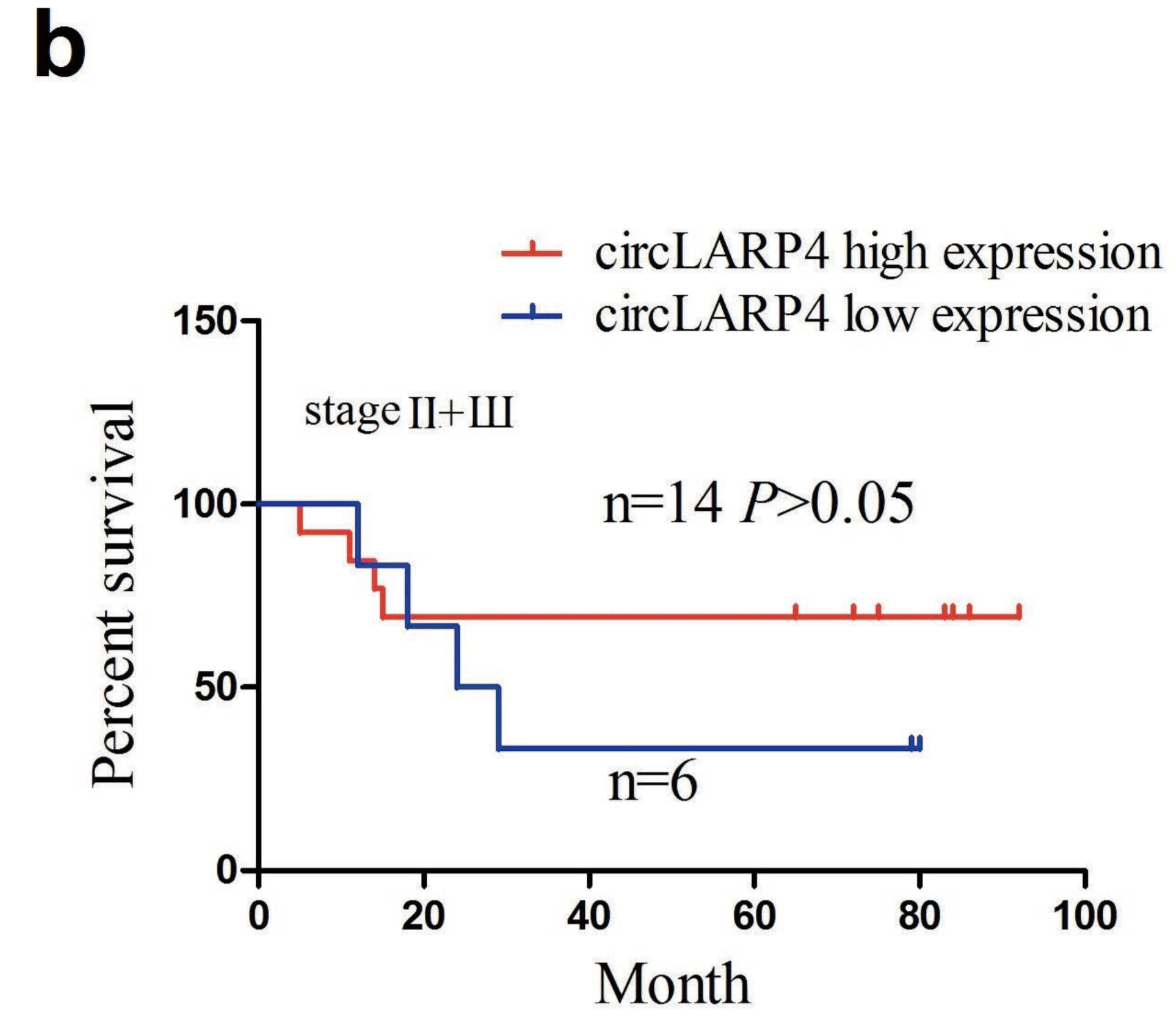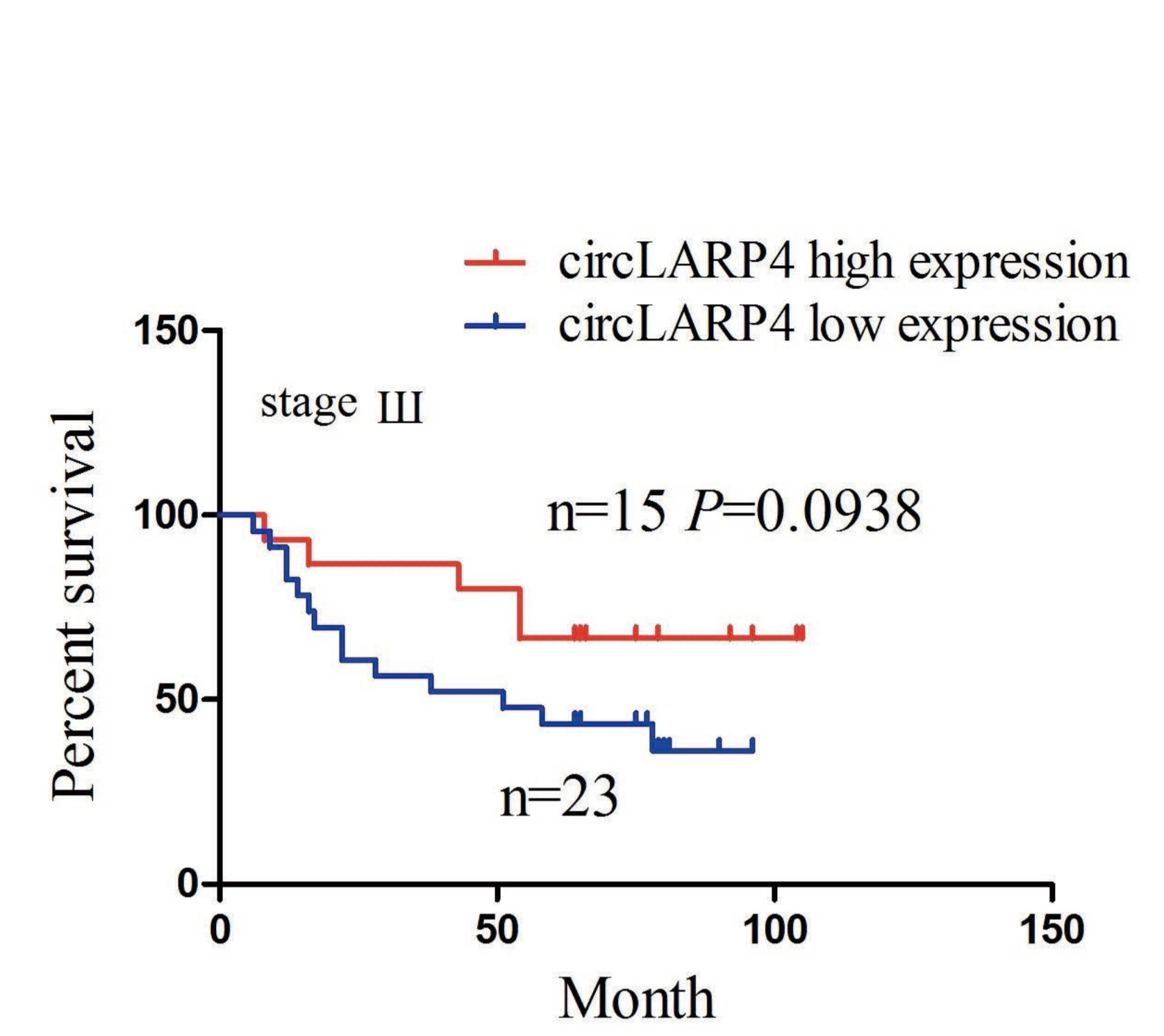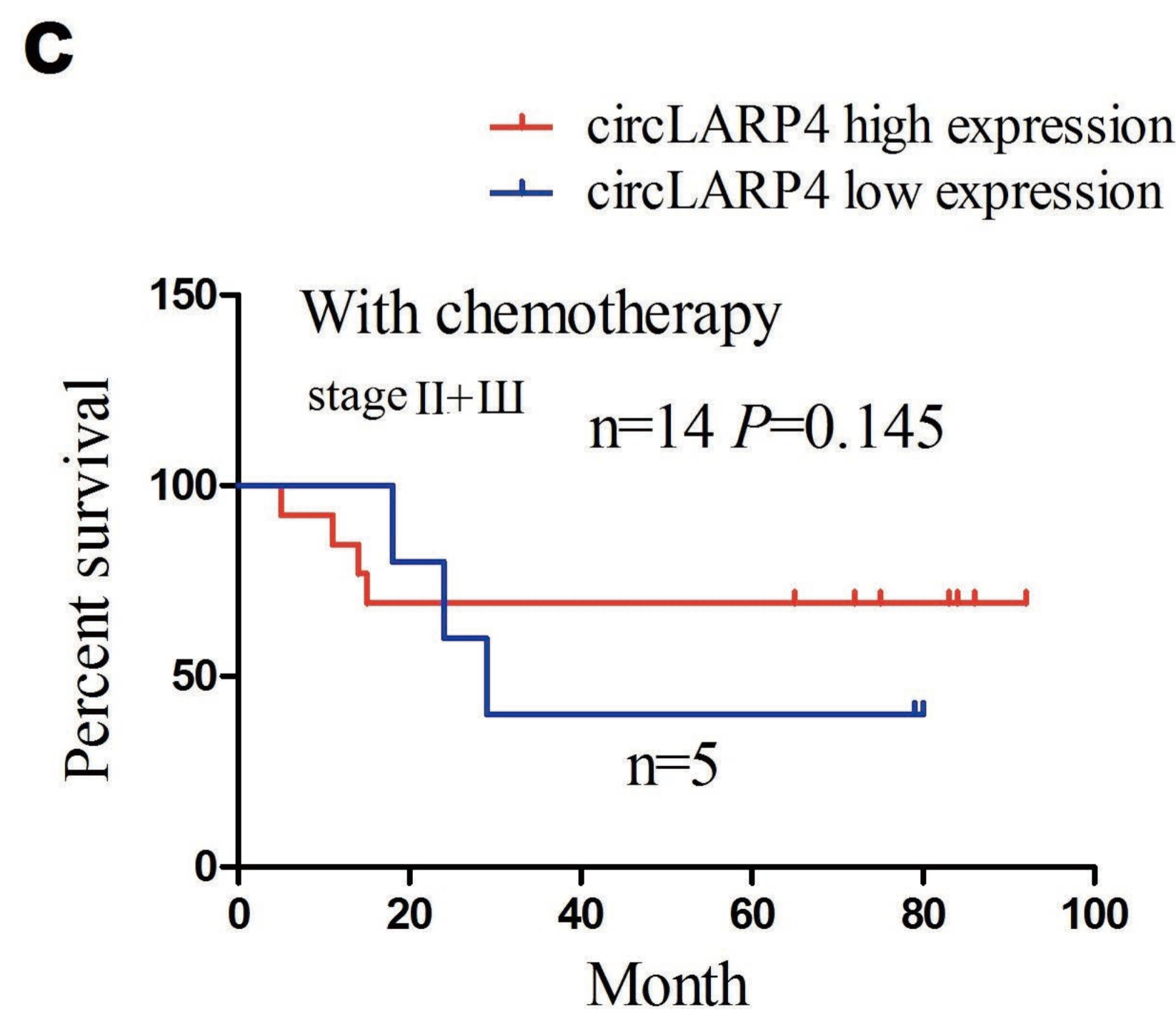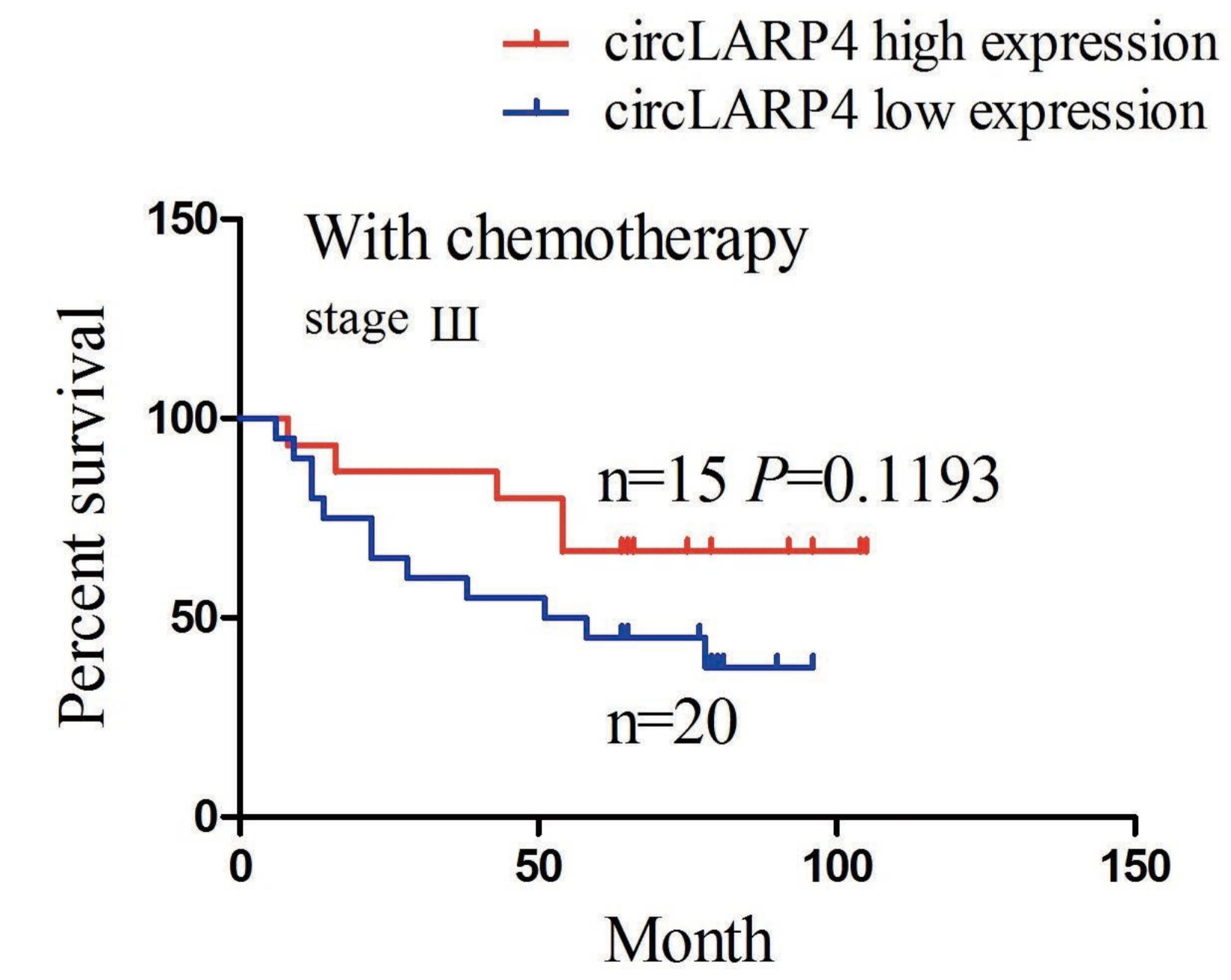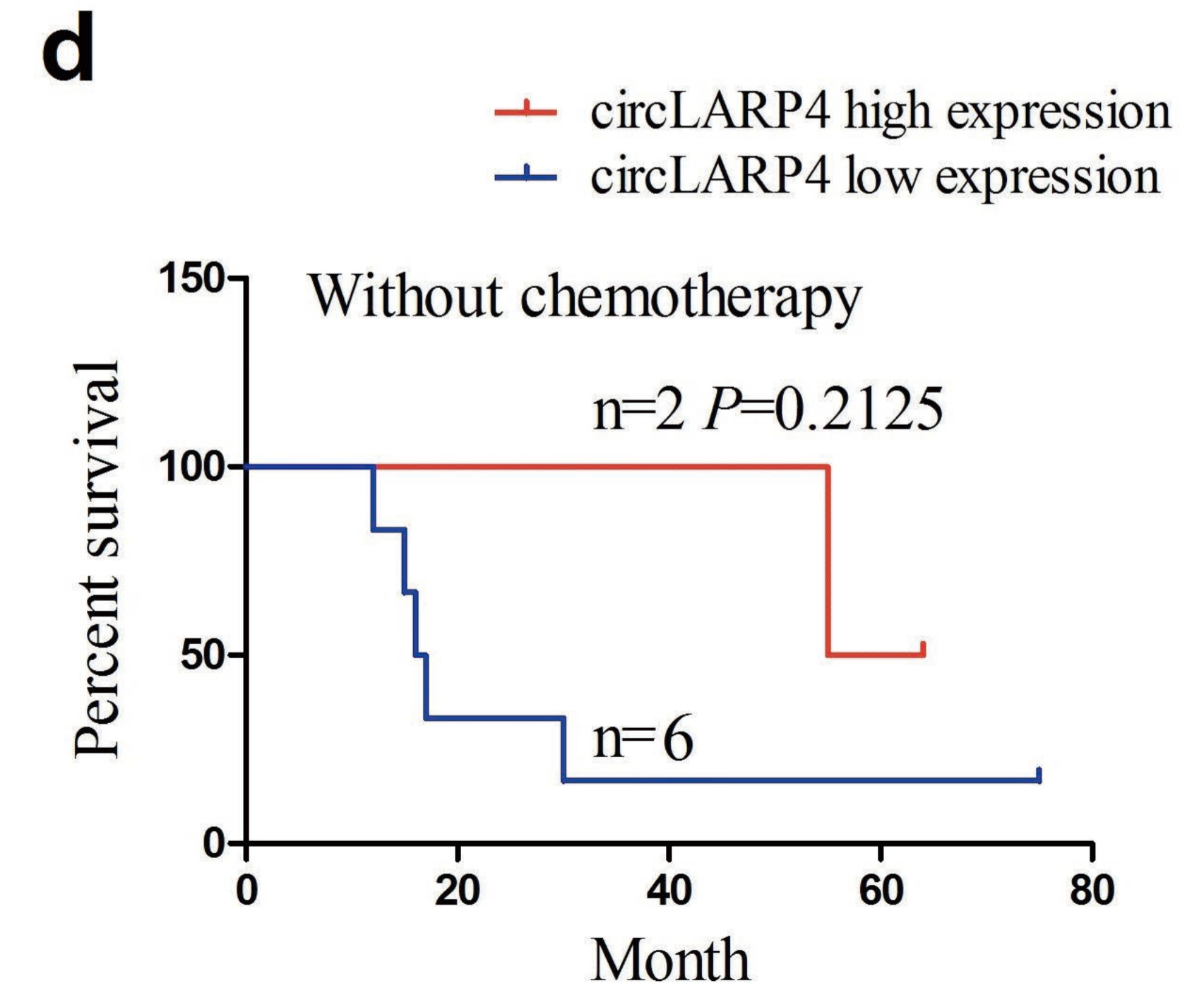

Supplement: Supplementary file 6 — Correlation of circLARP4 expression level with OS of GC patients. a Receiver operating characteristic (ROC) curve analysis of the cutoff value, sensitivity, specificity and AUC of circLARP4 in GC patients. b Kaplan-Meier analysis of the correlation of circLARP4 expression with OS of GC patients with stage II + III or stage III. c Kaplan-Meier analysis of the correlation of circLARP4 expression level with therapeutic outcomes of GC patients with stage II + III or stage III treated with adjuvant chemotherapy of oxaliplatin and 5-Fu. d Kaplan-Meier analysis of the correlation of circLARP4 expression level with therapeutic outcomes of GC patients without adjuvant chemotherapy. (PDF 3527 kb) [file 12943_2017_719_MOESM6_ESM.pdf]
