# Supplementary material for: Circular RNA_LARP4 inhibits cell proliferation and invasion of gastric cancer by sponging miR-424-5p and regulating LATS1 expression
Source: Mol Cancer. 2017 Sep 11;16:151. doi: 10.1186/s12943-017-0719-3 (PMC5594516; doi:10.1186/s12943-017-0719-3)
Supplement: Supplementary file 5 — The binding sites of circLARP4 with miRNAs. a Schematic representation of potential binding sites of miRNAs with circLARP4. b The effects of miR-424 mimic or inhibitor on the expression level of circLARP4 in HCG-27 or MKN-28 cell line indicated by qRT-PCR. c The binding sites of wild type or mutant circLARP4 3’UTR with miR-424.-5p. d qRT-PCR analysis of the expression levels of LATS1 and YAP after transfection with circLARP4 + miR-424 in HGC-27 cells or si-circLARP4 + miR-424 inhibitor in MKN-28 cells. e the luciferase activity of wild type LATS1 3’UTR was examined by co-transfection with miR-424 mimic + circLARP4 in HGC-27 cells. f the luciferase activity of wild type LATS1 3’UTR was detected by co-transfection with miR-424 inhibitor + si-circLARP4 in MKN-28 cells. *P < 0.05; **P < 0.01. (PDF 2681 kb) [file 12943_2017_719_MOESM5_ESM.pdf]

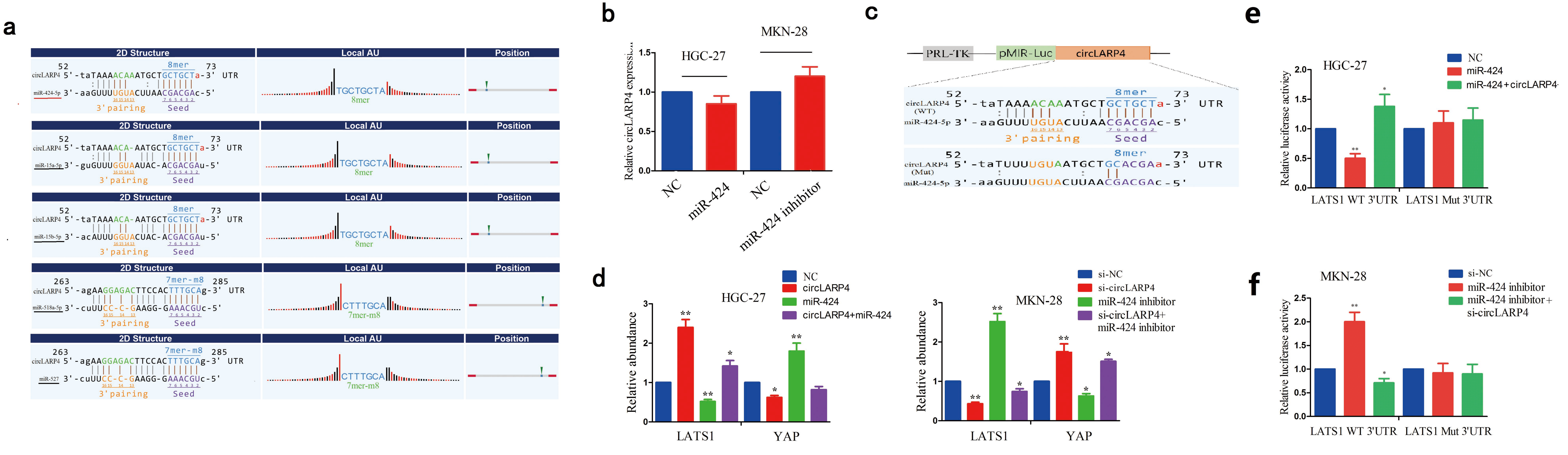

a
2D Structure
Local AU
Position
circLARP4 5'-taTAAAACAAATGCTGCTGCTa-3' UTR
miR-424-5p 3'-aaGUUUUGUACUUAACGACGAc-5'
8mer
3'pairing
Seed
TGCTGCTA
miR-15a-5p 3'-guGUUUGGUAAUAC-ACGACGAu-5'
miR-15b-5p 3'-acAUUUGGUACUAC-ACGACGAu-5'
circLARP4 5'-agAAGGAGACTTCCACTTTGCAg-3' UTR
miR-518a-5p 3'-cuUUCC-C-GAAGG-GAAACGUc-5'
miR-527 3'-cuUUCC-C-GAAGG-GAAACGUc-5'
7mer-m8
CTTTGCA
b
Relative circLARP4 expressi...
HGC-27
MKN-28
NC
miR-424
miR-424 inhibitor
c
PRL-TK
pMIR-Luc
circLARP4
circLARP4 (WT) 5'-taTAAAACAAATGCTGCTGCTa-3' UTR
miR-424-5p 3'-aaGUUUUGUACUUAACGACGAc-5'
circLARP4 (Mut) 5'-taTUUUUGUAATGCTGCACGAa-3' UTR
miR-424-5p 3'-aaGUUUUGUACUUAACGACGAc-5'
d
HGC-27
NC
circLARP4
miR-424
circLARP4+miR-424
Relative abundance
LATS1
YAP
MKN-28
si-NC
si-circLARP4
miR-424 inhibitor
si-circLARP4+ miR-424 inhibitor
e
HGC-27
NC
miR-424
miR-424+circLARP4
Relative luciferase activiey
LATS1 WT 3'UTR
LATS1 Mut 3'UTR
f
MKN-28
si-NC
miR-424 inhibitor
miR-424 inhibitor+ si-circLARP4
